# Efficient Solution‐Phase Synthesis of Sequence‐Defined Oligourethanes with Precise Chirality Control

**DOI:** 10.1002/marc.202500378

**Published:** 2025-06-22

**Authors:** Anuj Sharma, Tapendu Samanta, Joanna Cybińska

**Affiliations:** ^1^ Łukasiewicz Research Network – PORT Polish Center for Technology Development Wrocław Poland; ^2^ Department of Chemistry University of Wrocław Wrocław Poland

**Keywords:** chiral oligourethanes, one‐pot synthesis, sequence‐defined oligomers, sequence folding, structure‐property relationship

## Abstract

Nature relies on precisely defined macromolecules for complex biochemical processes with exceptional specificity and efficiency. To replicate these properties in synthetic systems, chemists have developed sequence‐defined macromolecules—polymers with absolute control over monomer sequence and structure, enabling tailored functions. However, their exploration in material science remains limited due to the challenges of synthesis, which is often low‐yielding and time‐consuming. To address this, we designed and synthesized Fmoc‐assisted stereo‐controlled sequence‐defined oligourethanes (SDOUs) in the solution phase. Our step‐economical synthesis employs a two‐step, one‐pot strategy, eliminating intermediate purification and achieving an average yield of >85% per step. The optimized protocol, using six modified chiral monomers, enables precise stereochemical and sequence control. Thermal analysis revealed that stereochemistry significantly influences thermal transitions, including glass transition, crystallization, and melting behaviors. Tandem mass spectrometry provided in‐depth sequencing analysis. We also demonstrated the post‐synthetic modification of the SDOUs with dansyl chloride and explored their photophysical properties, such as solvatochromism and aggregation. Circular dichroism analysis highlighted their unique structural and conformational features. This approach establishes scalable, efficient synthetic routes for stereochemically controlled sequence‐defined oligourethanes with diverse functional groups.

## Graphical abstract

A one‐pot, high‐yield strategy enables the solution‐phase synthesis of sequence‐defined, stereoregular oligourethanes using Fmoc chemistry. The resulting oligomers exhibit precise sequence and chirality control and allow post‐synthetic modification. Their structural and photophysical properties, including solvatochromism and aggregation behavior, are explored. This method provides a versatile platform for functional precision oligomers in advanced material applications.

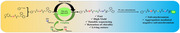

## 1 Introduction

Synthetic polymer materials that mimic the complexity and sophistication of natural biological systems have been a long‐standing challenge in materials science. Nature uses the primary sequence control of natural macromolecules, such as proteins, to fold into specific shapes and self‐organize into complex structures with various functions [1, 2]. Enzymes, as nature's most sustainable catalysts, exemplify this precision by possessing well‐defined structures with controlled sequences and chirality, enabling them to perform remarkable biochemical transformations essential for life [3, 4]. Emulating these properties in synthetic systems has led to the development of sequence‐defined polymers, a novel class of polymers characterized by precise control over monomer sequence and structure [5, 6]. These discrete sequence‐defined polymers mimic short peptides and hold significant potential for applications in catalysis [7, 8], information storage [9, 10, 11], drug delivery [5, 12], and self‐assembly [13, 14].

Recent advancements in polymer chemistry have led to the development of methods to synthesize uniform polymers with perfectly defined monomer sequences [15, 16]. These methods are usually based on iterative synthesis, which can be performed in solution [17, 18, 19, 20, 21] or on solid [22, 23] phase, allowing full control over monomer structure, sequence, and stereochemistry. However, the synthesis of sequence‐defined polymers faces several challenges. The use of solid support simplifies purification between steps but requires excess reagents and solvents, which can be both costly and environmentally unfriendly [24, 25, 26]. Moreover, the issues related to solid support, such as diffusion limitations and incomplete reactions remain unresolved. On the other hand, solution‐phase synthesis offers notable advantages over solid‐phase synthesis for sequence‐defined polymers. It enables larger scale reactions and typically achieves higher yields under a homogeneous environment. It allows for easier monitoring and precise control using techniques like high‐performance liquid chromatography (HPLC) and nuclear magnetic resonance (NMR). Further, it accommodates a broader range of reaction conditions and diverse monomers, facilitating the synthesis of complex polymer architectures with simplified purification techniques such as extraction and column chromatography [17, 27, 28]. Despite several advantages, the solution‐phase synthesis is cumbersome due to the need for purification between steps, leading to a reduction in overall yield. Consequently, there are few protocols that enable a scalable and sustainable way of making sequence‐defined polymers.

Polyurethanes are a versatile class of polymers characterized by the presence of urethane linkages (─NH─COO─) in their backbone. These are materials with a diverse spectrum of chemical and mechanical characteristics [29, 30]. Polyurethanes are extremely versatile and can be used in a wide range of applications, such as elastomers, adhesives, varnishes, flexible and stiff foams, and sealants. Synthetic sequence‐defined polyurethanes with precise monomer sequences and controlled chirality present a versatile structural framework that can be explored for different types of monomers, resulting in diverse structures, properties, applications, and functions [31, 32]. The carbamate functionality in these materials offers outstanding chemical and proteolytic stability, the capacity to pass through cell membranes, and the potential to regulate interactions with specific enzymes or receptors, rendering them appealing for biomedical purposes [22, 33]. Despite the promising potential of stereo‐regulated sequence‐defined polyurethanes, the developed materials are still far from achieving the advanced properties displayed by living systems. To approach such sophisticated functions, it is crucial to better understand the relationship between monomer sequence and polymer properties.

Considering the importance of sequence‐defined polyurethanes in material science, here we propose an easy, cost‐effective synthetic route for synthesizing sequence‐defined polymers with high yield and multi‐functionality using Fmoc deprotection chemistry. In this study, we developed an Fmoc‐assisted synthetic strategy for stereo‐regulated and sequence‐defined oligourethanes in solution‐phase. The work involves a step‐economical synthesis where both the deprotection and coupling steps are performed in a two‐step, one‐pot strategy without the need for intermediate purification, leading to a rapid and improved synthetic procedure. This method significantly reduces the overall reaction time and produces oligomers in high yield, making it a highly efficient approach for the synthesis of sequence‐defined oligourethanes. Our approach leverages a library of modified chiral monomers to explore the impact of designed chiral sequences on the thermal and photophysical properties of the oligomers. Moreover, the versatility of this methodology enables the incorporation of functional moieties, such as fluorophores, through post‐synthetic modifications.

## 2 Result and Discussion

### 2.1 Synthesis of Sequence‐Defined Oligourethanes (SDOUs)

The application of Fmoc protection‐deprotection chemistry has been widely utilized in solid‐supported and soluble‐support synthesis [34] of sequence‐defined polymers (SDPs). However, its application in solution‐phase synthesis has been less explored due to the challenges associated with purification after each iterative step, typically requiring multiple‐column chromatography or recrystallization processes. This study addresses these limitations by proposing a novel, scalable, efficient solution‐phase synthesis of stereo‐regulated sequence‐defined oligourethanes (SDOUs), utilizing Fmoc deprotection and coupling chemistry in a one‐pot approach.

**FIGURE 1:**
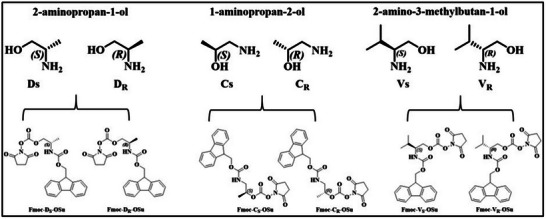
Monomer toolbox for synthesis of SDOUs.

To develop chirality and sequence‐defined oligourethanes, first, a series of chiral amino alcohols were modified to obtain desired monomers. The series of six modified monomers based on 2‐aminopropan‐1‐ol (Fmoc‐Ds‐OSu, Fmoc‐D_R_‐OSu), 1‐aminopropan‐2‐ol (Fmoc‐Cs‐OSu, Fmoc‐C_R_‐OSu) and 2‐Amino‐3‐methyl‐1‐butanol (Fmoc‐Vs‐OSu, Fmoc‐V_R_‐OSu) were synthesized. The ‘S’ and ‘R’ denoted the respective stereochemistry of the chiral amino alcohols. The monomer synthesis involves Fmoc‐protection of the amine group of amino alcohols by reacting it with 9‐Fluorenylmethyl‐succinimidyl carbonate (Fmoc‐OSu) followed by the activation of hydroxy group with N,N’‐disuccinimidyl carbonate (DSC). The synthetic protocol of monomers is represented in Figure S1 and the monomer toolbox is given in Figure 1. This process resulted in the formation of a library of six modified chiral monomers, which could be used for iterative deprotection‐coupling strategies to produce chiral sequence‐defined oligourethanes (SDOUs).

**SCHEME 1:**
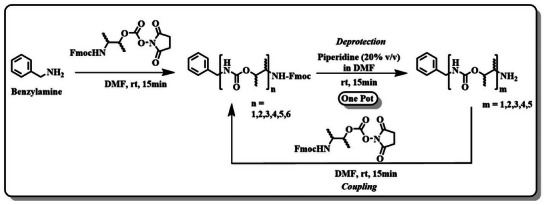
General synthetic scheme for chirality and sequence‐defined oligourethanes.

Toward the synthesis of SDOUs, we first confirmed the formation and purity of a total of six chiral monomers using ^1^H NMR spectroscopy analysis. The synthesis of SDOUs was initiated by reacting benzylamine with a desired chiral monomer in dimethylformamide (DMF). The reaction proceeded through a substitution mechanism, and complete consumption of the monomer was confirmed through reverse‐phase high‐performance liquid chromatography (HPLC) within 15 min. The resulting 1‐mer product (**SC1**, **RC1,** and **SRC1**) contained a benzyl group on one end and a Fmoc‐protected amine on the other. After the reaction, liquid‐liquid extraction with water and ethyl acetate was performed, effectively removing unreacted benzylamine and the N‐hydroxysuccinimide (NHS) byproduct, yielding pure 1‐mer. To propagate the oligomer synthesis, Fmoc deprotection of 1‐mer was carried out using 20% (v/v) piperidine in DMF, which was completed within 15 min as confirmed by HPLC. The reaction mixture was thoroughly dried using a rotary evaporator and co‐evaporated with ethyl acetate to remove excess piperidine. After drying, the next modified monomer was added to the same reaction pot in a minimal amount of DMF, initiating the coupling reaction to produce the 2‐mer SDOUs (**SC2**, **RC2,** and **SRC2**) in quantitative yield, which was also completed within 15 min as confirmed by HPLC. Upon completion of the coupling reaction, N‐hydroxysuccinimide (NHS) was efficiently removed via liquid–liquid extraction. To further purify the product, the crude was subjected to precipitation in hexanes, resulting in the removal of the Fmoc‐piperidine adduct and dibenzofulvene (DBF) as soluble impurities. The pure 2‐mer was obtained as a white solid precipitate, demonstrating effective separation and purification. Additionally, this one‐pot deprotection‐coupling approach minimized solvent and time consumption while preserving the reaction yield. This one‐pot deprotection‐coupling strategy combined with hexane washing was repeated iteratively to introduce each desired monomer and synthesized pure SDOUs up to hexamers (6‐mers) in high yields. Using this method, three sequences comprising S stereo‐configured monomers (**SC6**), R stereo‐configured monomers (**RC6**); and an alternate mixture of S and R stereo‐configured monomers (**SRC6**) were successfully synthesized in six steps with the overall synthesis yields of 67%, 70%, and 72%, respectively. The process achieved minimal yield loss during washing and extraction steps, as demonstrated in Tables S1–S3. The general synthetic scheme for the sequence is depicted in Scheme 1 and detailed synthetic schemes for each stereo‐specific sequence are depicted in Figures S2–S4. The progression of each coupling step was monitored using reversed‐phase HPLC and the obtained spectra are shown in Figures S5–S7. All obtained sequences exhibited a uniform structure with high purity, as demonstrated by NMR (Figures S8–S54), and HRMS (Figures S55–S57). The synthetic scheme for **SRC6**, accompanied by ^1^H NMR analysis of each sequence is presented in Figure S58, highlighting the successful synthesis and structural confirmation of the target sequence.

Spectroscopic analysis and HPLC techniques confirmed the successful sequencing of respective chiral monomers with high precision along with high purity, and quantitative yield through a rapid and easy but promising synthetic strategy. Moreover, this synthetic strategy overcomes the obstacles of the liquid‐phase Fmoc deprotection process to develop a new route to obtain SDOUs. Although the synthesized hexamers (**SC6**, **RC6**, and **SRC6**) share the same molecular weight (Mw) of 992.137, the distinct arrangements of chiral monomers within their sequences can significantly influence their physical and chemical properties.

### 2.2 Thermal Properties

The initial goal was to evaluate the thermal behavior of the oligomers. Towards this, the thermal characteristics of the sequence‐defined oligourethanes are assessed using thermogravimetric analysis (TGA) and differential scanning calorimetry (DSC) to understand the impact of chirality on their thermal stability and transitions. All three sequences, with identical molar masses, showed excellent thermal stability in TGA measurements as shown in Figure S59. The degradation temperatures corresponding to 10% weight loss were 216 °C, 213 °C, and 212 °C. The minor differences in thermal stability can be attributed to the identical molecular weight and similar overall chemical composition, with variations primarily due to the chirality of monomers.

**FIGURE 2:**
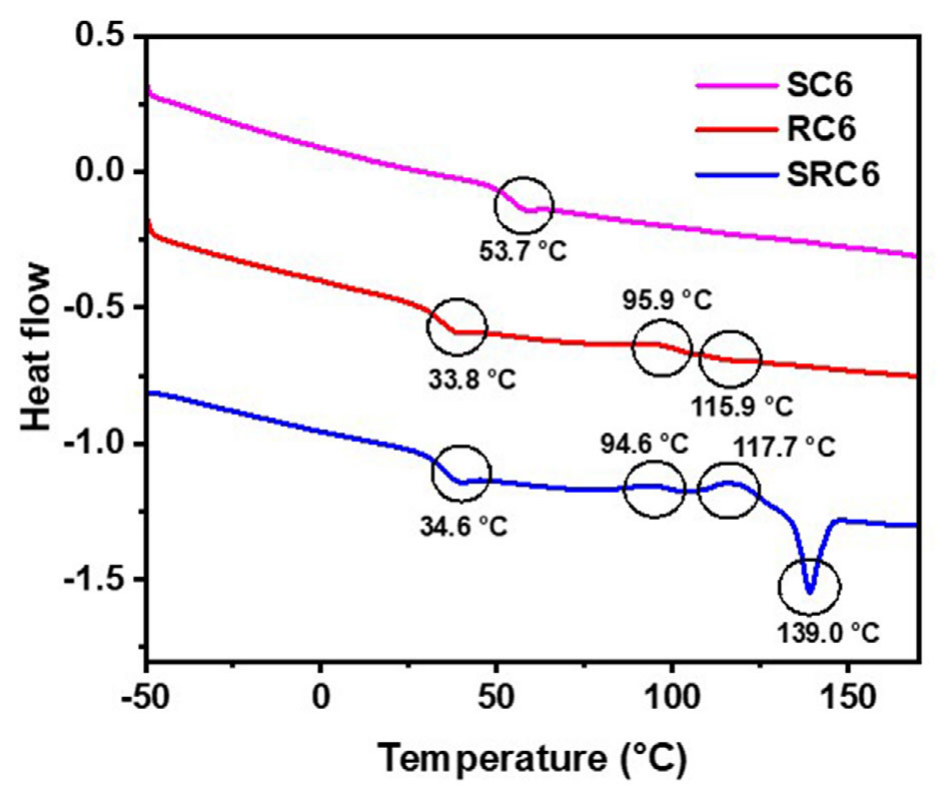
DSC spectra of **SC6**, **RC6**, and **SRC6** sequences.

DSC analysis provided deeper insights into the thermal transitions of the sequences. The obtained spectra are shown in Figure 2 and their corresponding data are presented in Table S4. In the second heating cycle, **SC6** exhibited a glass transition temperature (Tg) at 53 C indicating amorphous behavior of the sequence. In contrast, **RC6** demonstrated partial recrystallization and retained crystalline phases during second heating with a crystallization temperature (Tc) at 96 °C, and a melting temperature (Tm) at 116 °C, suggesting enhanced thermal organization due to the respective chirality. Interestingly, **SRC6** displayed the most complex behavior, with the emergence of multiple crystallization transitions during the second heating cycle with two distinct crystallization transitions at 95 °C and 118 °C, and a melting temperature at 139 °C (Figure 2). This behavior is likely influenced by alternating S and R chirality introducing irregular packing and steric interactions. These findings highlight that chirality significantly influences the crystallinity and thermal transitions of sequence‐defined oligourethanes. Further studies are necessary to understand the role of alternating chirality in promoting these unique thermal properties.

### 2.3 Tandem Mass (MS/MS) Analysis of the SDOUs

**FIGURE 3:**
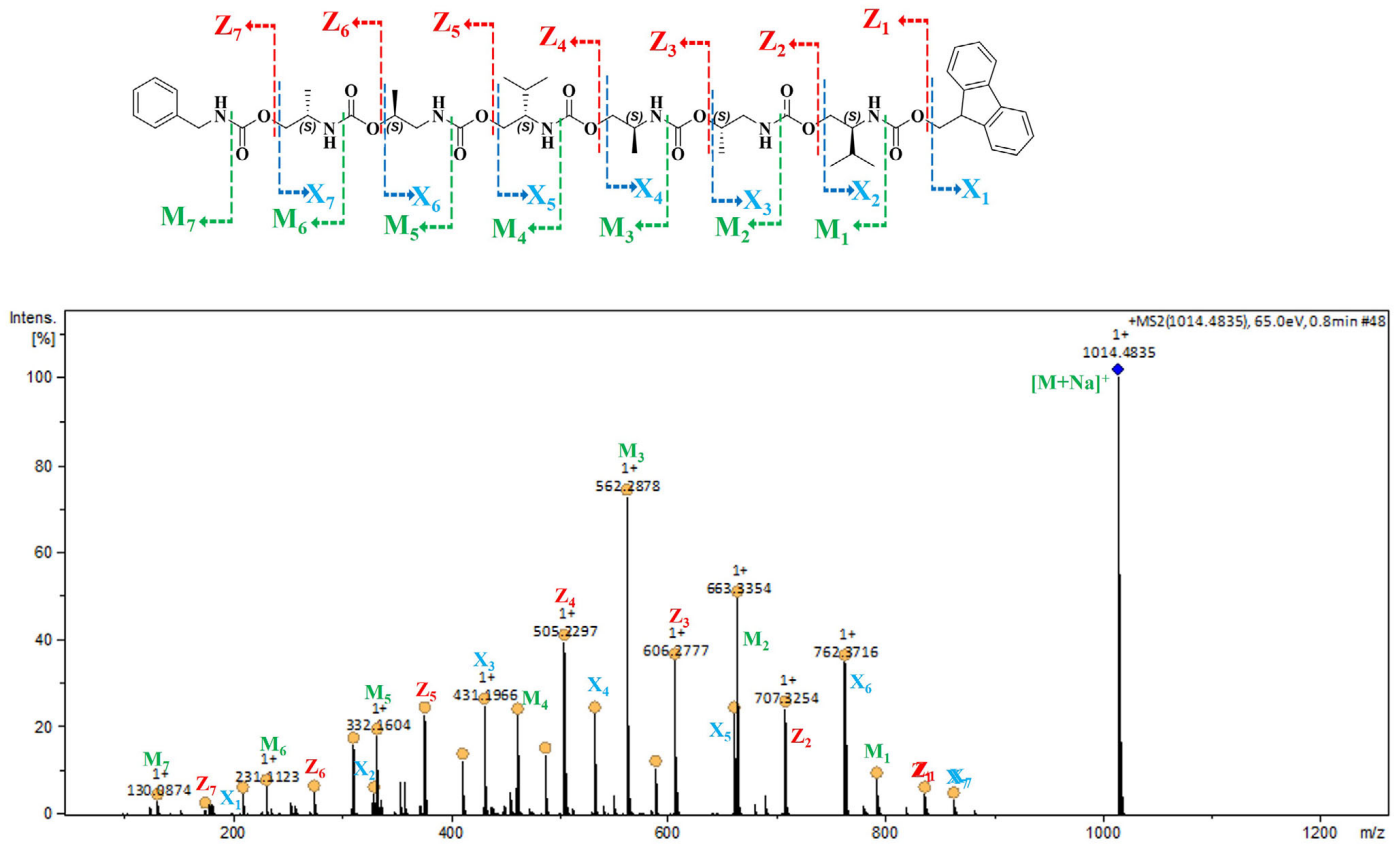
Tandem MS spectrum of **SC6**, illustrating the fragmentation pattern of the [M+Na]^+^ ion peak, with all fragment peaks corresponding to sodiated ions.

To determine the sequencing pattern in SDOUs, tandem mass spectroscopic (MS/MS) technique was employed, a versatile analytical method widely utilized for structural elucidation, quantification, and polymer sequencing. The primary objective was to verify the presence of monomer fragments in the sequences as per the order of synthesis carried out to prepare SDOUs. The MS/MS analysis of **SC6** with a molecular ion peak at [M+Na]^+^ = 1014.48 revealed three distinct fragmentations in each step. The first fragment (X_n_) which is an eliminated product formed due to the cleavage of C─O bond and the hydrogen of alpha carbon of carbamate bond leaving the second fragment (Z_n_) which is in R‐NH(CO)OH form. The final fragment (M_n_) is due to the release of CO_2_ from the R‐NH(CO)OH segment to give R‐NH_2_ ion. For **SC6**, MS/MS analysis revealed the pattern of the sequence where the end of the sequence consists of Fmoc group followed by valinol, 1‐aminopropan‐2‐ol, 2‐aminopropan‐1‐ol, valinol, 1‐aminopropan‐2‐ol, 2‐aminopropan‐1‐ol and finally the benzyl amine groups. From this tandem MS analysis study, it is clear that there are 21 fragments (A_1_‐A_7_, Z_1_‐Z_7_ and M_1_‐M_7_) for **SC6** (Figure 3). Detailed fragmentation patterns are shown in supporting information (Figure S60). Similar fragmentation behavior was observed for **RC6**, confirming the consistency of the sequence‐specific cleavage pathways. (Figure S61). Notably, **SRC6** exhibited a fragmentation pattern reflecting its alternating S and R chirality, as expected from the synthetic route. In the case of **SRC6**, the observed sequencing is Fmoc, valinol, valinol, 1‐aminopropan‐2‐ol, 1‐aminopropan‐2‐ol, 2‐aminopropan‐1‐ol, 2‐aminopropan‐1‐ol and benzylamine (Figure S61). These findings provide robust evidence that the oligourethanes were synthesized with precise sequence control and stereochemical fidelity.

### 2.4 Post‐Synthetic Modification of Sequence‐Defined Oligourethanes

**SCHEME 2:**
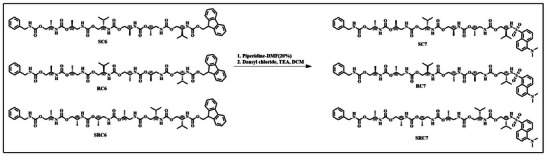
Synthetic strategy of Dansyl terminated SDOUs.

According to our synthetic strategy, all the SDOUs terminate with an Fmoc‐protected amine group. This Fmoc protection strategy provides a modular design where deprotection exposes a free amine for further chain extension. This approach enables the synthesis of longer sequences with tunable functionality. To establish this claim, we functionalized each SDOU with Dansyl chloride, a fluorescent dye. This post‐synthetic modification was performed via a two‐step synthetic process, as depicted in Scheme 2. Dansyl incorporation into the SDOUs is a straightforward and efficient process with excellent yield.

**FIGURE 4:**
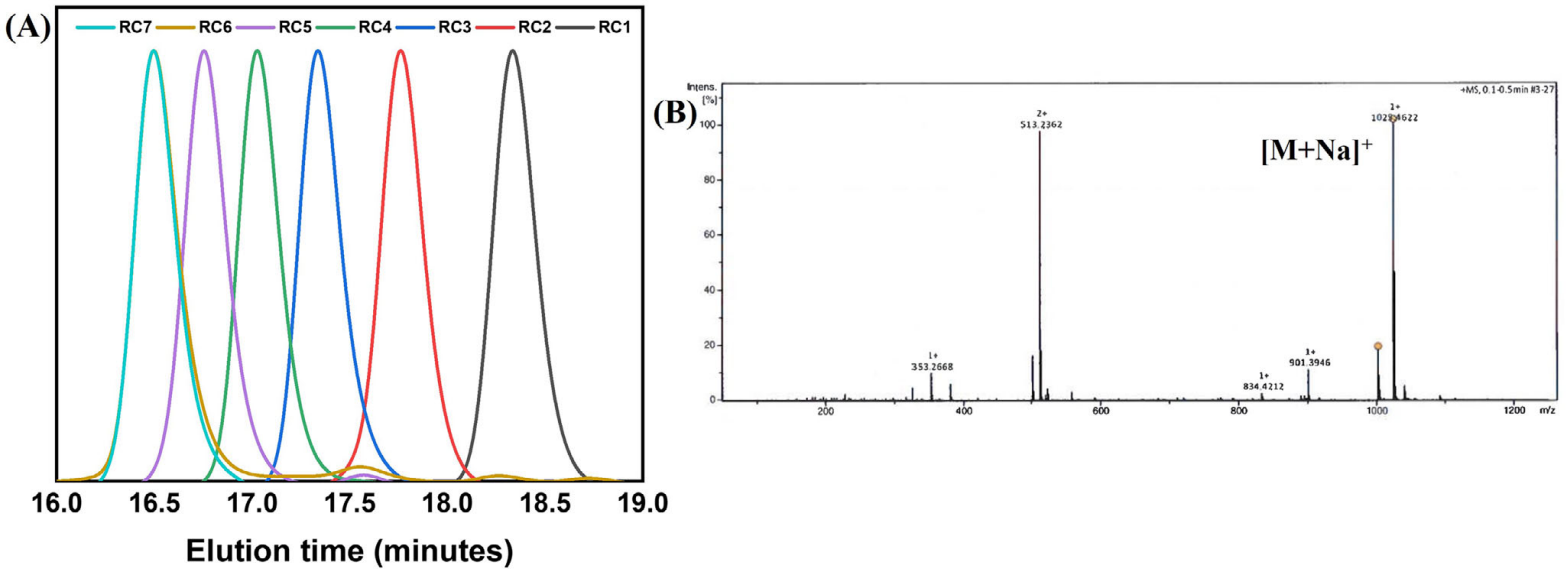
(A) SEC analysis of R stereospecific SDOU and (B) ESI‐MS analysis of **RC7** (Calculated [M+Na]^+^: 1025:47, observed [M+Na]^+^: 1025.46).

Size exclusion chromatography (SEC) is an excellent tool to monitor the sequence's progression. Each monomer incorporation is responsible for the increased molecular weight of the sequences and it could be tracked by SEC analysis. Here we performed SEC analysis for each step during the SDOU synthesis. For the R stereospecific configured sequence (**RC7**), SEC analysis showed an expected lower elution time shift after each monomer attachment to the sequence. This suggests perfect sequencing and its progression throughout the synthesis (Figure 4A). The formation of **RC7** was further confirmed by ESI‐MS analysis (Figure 4B) with a molecular ion peak at [M+Na]^+^: 1025.46 (calculated [M+Na]^+^: 1025.47). Similar SEC traces and ESI‐MS were observed for S stereospecific (**SC7**) and mixed (**SRC7**) SDOUs, as shown in Figures S63 and S64. All these SEC and ESI‐MS results suggest successful sequencing of the SDOUs with high purity. This post‐synthetic modification further demonstrated the modularity and functional flexibility of the sequences. Thus, any kind of desired functionalization of the SDOUs can be achieved using our synthetic strategy for diverse applications.

### 2.5 Photophysical Studies of the Dansyl Terminated SDOUs

**FIGURE 5:**
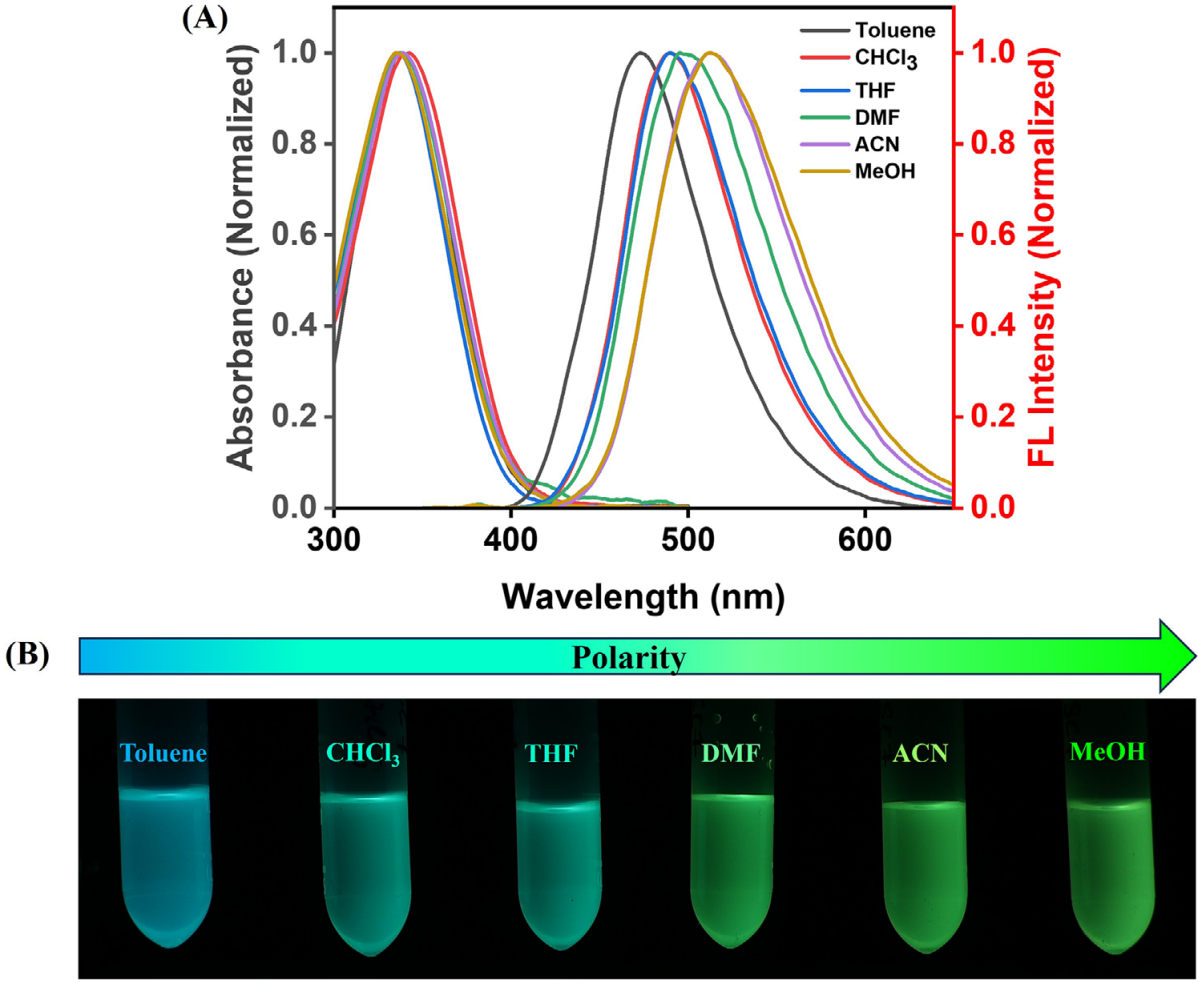
(A) Normalized absorbance and emission spectra of **SC7** in different solvents; (B) Pictorial representation of **SC7** solutions in different solvents under hand‐held UV light. (λ_ex_ = 340 nm).

To investigate the photophysical properties of SDOUs, we functionalized them with dansyl chloride to obtain dansylamide analogs. Dansyl derivatives exhibit polarity‐dependent fluorescence properties, making them useful probes for environmental sensitivity. It is well established that dansylamide‐like derivatives have a complex excited‐state nature which influences the emission properties with solvent polarity [35, 36]. This phenomenon has been extensively studied in related systems, such as 1‐(dimethylamino)‐5‐naphthalenesulfonic acid and its derivatives [37, 38]. Based on this knowledge, we performed solvent‐dependent absorbance studies followed by their respective emission analysis. Absorbance studies of **SC7** revealed two distinct absorbance bands around 255 and 340 nm in various solvents like toluene, chloroform, THF, DMF, acetonitrile, and methanol (Figure 5A). The observed ∼255 nm corresponds to π → π* transitions of the benzyl group within the sequence, while the ∼340 nm peak is a characteristic of the π → π* transitions of the dansyl segment. Comparable absorbance bands were observed for **RC7** and **SRC7** sequences as shown in Figures S65 and S66. Notably, no significant changes were observed in absorbance with varying solvent polarity. This is attributed to the unaltered ground states of dansyl‐coupled sequences upon change of solvent polarity. Upon excitation at 340 nm, **SC7** exhibited strong emission at around 473 nm in toluene, which gradually red‐shifted upon increasing the polarity of solvents from chloroform to methanol. With increasing solvent polarity, **SC7** exhibited large stokes shifts (in the range of 135–177 nm, as shown in Table S5) between absorbance and emission maxima, which highlights its applicability in bioimaging, sensing, etc. The fluorescence studies showed that the emission maxima of **SC7** were gradually red‐shifted (473 to 513 nm) with changing solvent polarity (Figure 5B). This spectral shift of **SC7** in different solvents was accompanied by a visible change in emission color from cyan in non‐polar solvent to greenish‐yellow in polar solvent, under UV light illumination. A similar red‐shifted emission behavior was observed for **RC7** and **SRC7** (Figures S67 and S68). The observed solvatochromism can be explained by solvent‐induced excited state relaxation, which is a characteristic phenomenon of dansyl derivatives due to their strong charge‐transfer (CT) properties in the excited state [39].

### 2.6 Aggregation Studies of SDOUs

**FIGURE 6:**
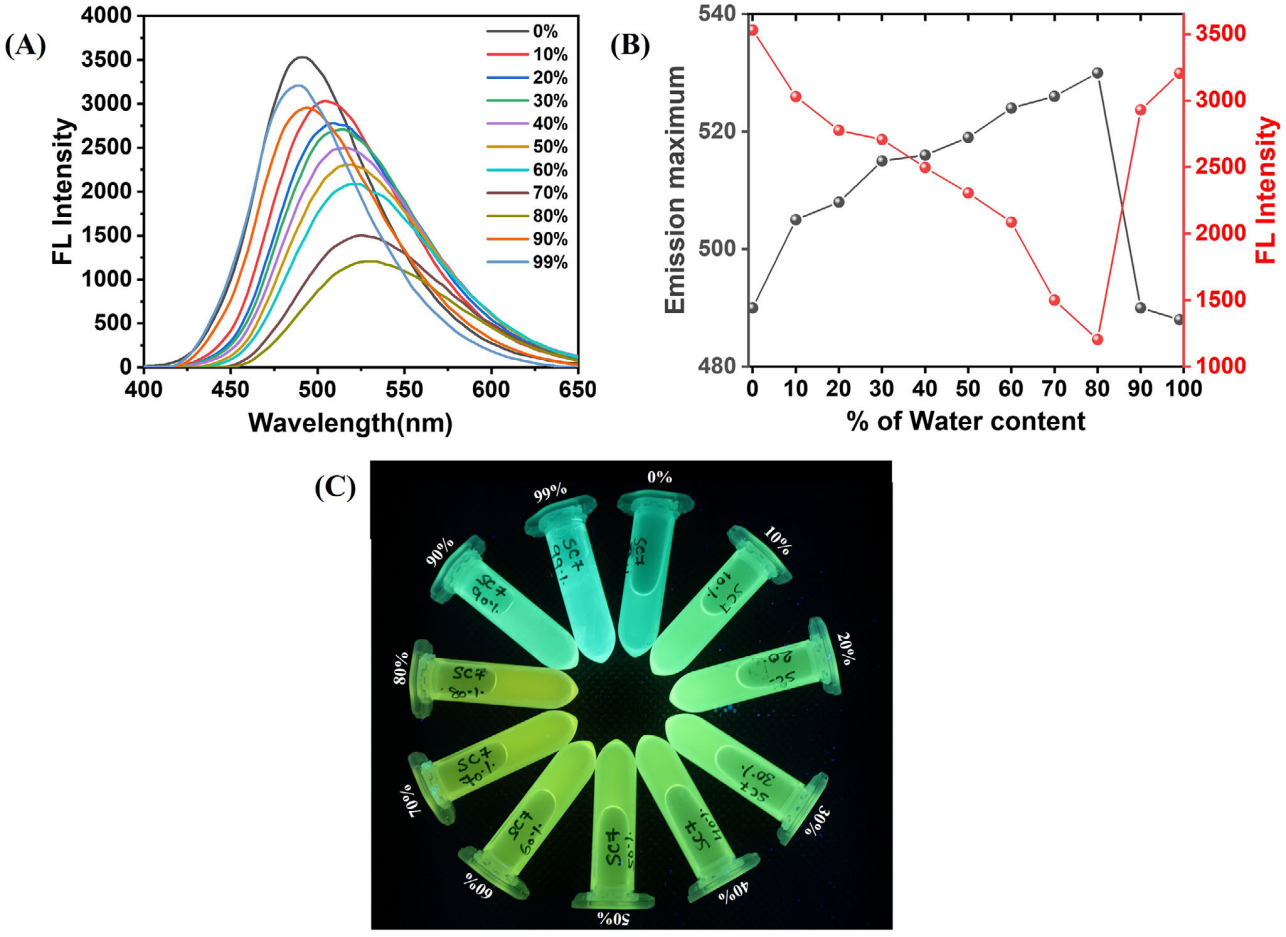
(A) Fluorescence spectroscopic study of **SC7** (10 µm) with varying water content in THF; (B) Plot of emission maxima and emission intensity as a function of water content for **SC7**; (C) Pictorial representation of **SC7** solutions for different THF‐water content under hand‐held UV light.

To further evaluate the photophysical properties of the SDOUs, we monitored their emission behavior as a function of water content in the THF medium. In the case of the **SC7** sequence, upon excitation at 340 nm, a gradual red shift of emission maxima along with decreased intensity was observed with increasing water content up to 80%. However, a sudden increase in emission intensity accompanied by a blue shift of emission maxima was recorded at 90%–99% water content (Figure 6A). The change of emission maxima with intensity corresponds to increasing water content in the THF medium is depicted in Figure 6B, where the change is clearly visible for better understanding. This initial redshift followed by a drastic blue shift in emission maxima for **SC7** was also observed under the UV light, where the color of solutions shifted toward yellow with increasing amounts of water up to 80% and then returned to cyan at 90%–99%. A similar observation was noted for the **RC7** sequence (Figure S69). Interestingly, **SRC7** showed a different spectral behavior in response to water content variations. An increase in emission intensity along with the red shift in emission maxima till 20% water content was observed, a trend differing from the other two sequences. From 30% to 80% water content, a gradual decrease in emission intensity along with red‐shifted emission maxima was observed for **SRC7**, aligning with the trend for the other two SDOUs. Similarly, an obvious blue shift with increased emission intensities was obtained at 90%–99% water content. Here, the emission color transitioned from cyan to greenish‐yellow and then reverted to cyan, consistent with other sequences (Figure S70).

This observed shift in emission maxima and color could be attributed to two reasons, the solvent polarity effect on SDOUs or hydrophobic–hydrophilic interaction between dansyl moiety and its surroundings. Two key emission features were observed for all SDOUs with changing water content in the THF medium. The first was a gradual red shift in the emission band for SDOUs with decreased intensity up to 80% of water content. The second was a hypsochromic shift in the emission bands with increased intensity at 90% and 99% water content. Notably, at 99% of water, the emission maxima for each SDOUs closely resembled those in pure THF. This solvent‐dependent change in emission behavior up to 80% water content could be explained by excited state stabilization of the charge‐transfer state due to the increased solvent polarity. On the other hand, the drastic blue shift at 90% and 99% water content was observed which could be unlikely to be caused by hydrogen bonding with water as observed by Sahoo et al [40]. and Barik et al [41]. However, the emission spectral pattern of the SDOUs with changing water content differs from that observed by Sahoo et al., who reported a gradual hypsochromic shift in emission spectra upon the increase of water content in a binary solvent system [40]. Thus, hydrogen bonding mediated blue‐shift in emission bands for the SDOUs at 90%–99% water content in THF can be ruled out.

To better understand the observed emission behavior, we considered the hydrophobic‐hydrophilic interactions between SDOUs and the surrounding medium. Dansyl analogs tend to exhibit red‐shifted emission in hydrophilic or more polar mediums and reverse emissive behavior in hydrophobic or less polar environments [39, 42]. It is well reported that dansyl derivatives tend to show increased emission intensity with blue‐shifted emission bands in the hydrophobic environment or cavity [39]. In the case of our SDOUs, the initial red shift of fluorescence from cyan to greenish‐yellow color up to 80% water content could be due to the increase in a hydrophilic environment in the medium and the onset of aggregation formation. In this range, aggregation likely begins but the formed aggregates remain loose or partially solvated, allowing some interaction between the dansyl groups and the surrounding polar environment. This interaction leads to the red‐shifted (greenish‐yellow) fluorescence, driven by the charge‐transfer character of the excited state. On the other hand, at 90%–99% water content, a drastic blue shift of the emission band toward the cyan region with increased emission intensity was observed, which resembles pure THF. This could be achieved by the formation of more compact, well‐defined, and uniform aggregates of the SDOUs. Due to the formation of well‐defined aggregates, the dansyl segments are expected to be buried in a hydrophobic core thus exposing it to a hydrophobic microenvironment. This hydrophobic microenvironment leads to a blue‐shifted emission band along with increased intensity.

To support this hypothesis, we conducted dynamic light scattering (DLS) studies for **SC7**, **RC7,** and **SRC7** in different water‐THF mediums. For **SC7**, we observed two distinct particle sizes in DLS up to 80% water content with improved polydispersity index (PDI) values (Figure S71A–D). This suggested the formation of aggregates that are not compact or partially solvated, thus leading to the hydrophilic interaction of the dansyl segment with water. Interestingly, at 90% and 99% water content, we observed more monodispersed particles with excellent PDI values (Figure S71E,F), further supporting the predominance presence of compact aggregates. This compact aggregation pattern supports the drastic blue‐shifted emission and increased intensity of **SC7**. The particle size variations along with the PDI values are depicted in Table S6. For **RC7** and **SRC7**, a slightly different pattern in particle sizes was observed in DLS studies (Figures S72 and S73), where we observed a monodispersed particle pattern from 80% water content for both the sequences, while the overall remained consistent with **SC7**. The detailed DLS measurement parameters for **RC7** and **SRC7** are depicted in Tables S7 and S8, respectively.

**FIGURE 7:**
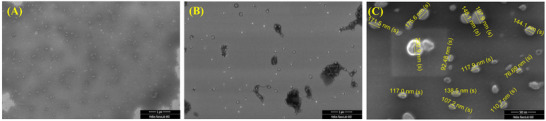
FESEM analysis of **SRC7** in (A) 50% water‐THF medium, (B) 99% water‐THF medium, and (C) 99% water‐THF medium with diameter measurements.

Field emission scanning electron microscopy (FE‐SEM) analysis was conducted to complement the DLS results for all the SDOUs. The morphological behavior of **SC7**, **RC7**, and **SRC7** was analyzed in both 50% and 99% water‐THF mixtures. For **SRC7**, an irregular particle size distribution and morphology were observed in the 50% water‐THF mixture (Figure 7A), aligning with the DLS results, where a high PDI value indicated the presence of two distinct particle populations. In contrast, **SRC7** exhibited remarkable uniformity in size and a well‐defined spherical morphology in the 99% water‐THF mixture, as evident in FE‐SEM images (Figure 7B,C). The observed spherical morphology of **SRC7** in the 99% water‐THF system strongly supports our hypothesis of hydrophobic‐hydrophilic interactions driving the negative solvatochromism. This behavior can be attributed to the exposure of the dansyl unit to a hydrophobic microenvironment within the self‐assembled particle core. A similar self‐assembly pattern was observed for **SC7** and **RC7** in FE‐SEM analysis (Figure S74), further reinforcing our hypothesis. The self‐assembly process is likely driven by hydrophobic‐hydrophilic interactions between the dansyl‐appended oligourethanes and water molecules, ultimately leading to the unique emission properties observed in SDOUs.

The synthesized dansyl‐appended SDOUs exhibited intriguing self‐assembly behavior in the water‐THF medium, leading to remarkable emission properties. This self‐assembly, driven by hydrophobic‐hydrophilic interactions, plays a crucial role in the observed negative solvatochromism. The formation of well‐defined spherical morphologies in highly aqueous environments, as confirmed through FE‐SEM analysis, further supports the controlled aggregation of these structures. Given their unique emission characteristics and organized self‐assembled nature, SDOUs hold significant potential for imaging studies in biomedical applications. Their ability to form stable, fluorescent nanoassemblies in aqueous environments makes them promising candidates for advanced bioimaging and diagnostic tools.

### 2.7 Exploring Oligomer Conformations Through Circular Dichroism

**FIGURE 8:**
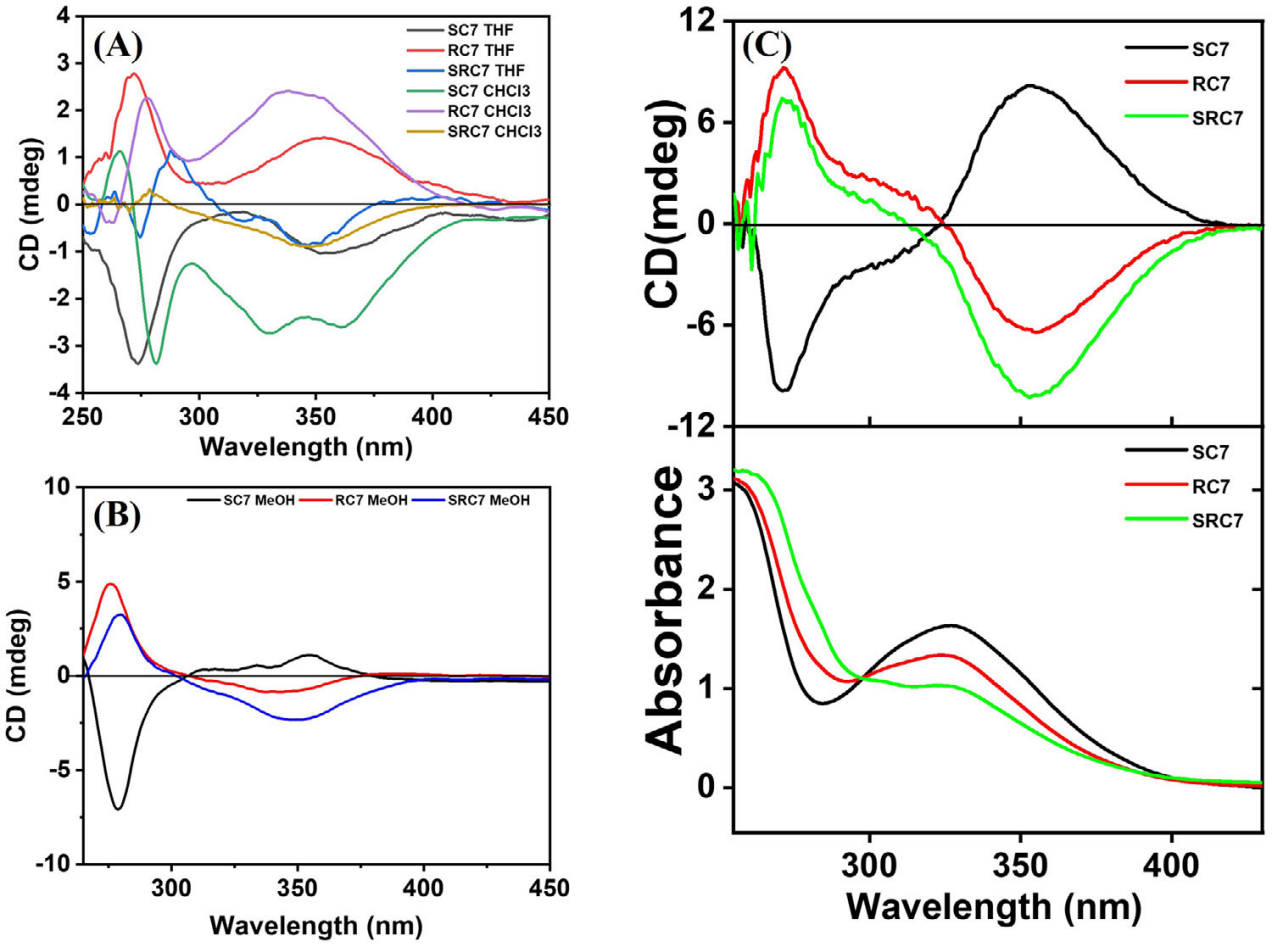
CD analysis of the SDOUs (50 µm) in (A) CHCl_3_ and THF, (B) MeOH, and (C) TFE along with corresponding absorption spectra.

Chiral monomers are the fundamental building blocks of the synthesized SDOUs, making it essential to investigate their structural and conformational properties. Circular dichroism (CD) analysis was conducted to assess these attributes in varying polarity solvents including chloroform (CHCl_3_), tetrahydrofuran (THF), methanol (MeOH), and 2,2,2‐trifluoroethanol (TFE). The obtained CD spectra are shown in Figure 8. All SDOUs exhibit two absorbance maxima around 255 and 340 nm, where the latter peak corresponds typically to the dansyl unit, leading to the expectation of two corresponding CD bands. The CD spectra in non‐polar solvents (CHCl_3_ and THF) reveal two negative Cotton bands for the S‐stereospecific oligomer (**SC7**) at around 281 and 338 nm, whereas the R‐stereospecific oligomer (**RC7**) showed two positive bands at the same wavelengths, confirming their enantiomeric nature (Figure 8A). This opposite behavior is characteristic of enantiomers, which generate CD signals of the same magnitude but opposite signs due to their mirror‐image structures. A slight blue shift to around 272 nm and 353 nm in THF suggests a minor solvent‐induced effect. The solvent‐independent nature of the bands indicates that the electronic transitions responsible for the Cotton effect are intrinsic to the chiral configurations of the oligomers [43]. These results highlight the stereospecificity and optical activity of the oligomers. The CD analysis of **SRC7** in both CHCl_3_ and THF revealed a weak negative band around 350 nm (Figure 8A), which can be attributed to the presence of an equal number of S and R‐configured monomeric units, leading to near cancellation of optical activity.

Circular dichroism analysis in polar solvents reveals significant conformational and structural changes. In MeOH, **SC7** exhibited a positive band at ∼355 nm and a negative band at ∼275 nm, while **RC7** showed an inverted pattern, indicating opposite chiral preferences (Figure 8B). **SRC7** displays a spectral profile similar to **RC7** but with increased intensity, suggesting a dominant R‐stereocenter influence near the dansyl moiety. To further investigate solvent‐induced structural rearrangements, CD analysis was conducted in 2,2,2‐trifluoroethanol (TFE), a solvent known for stabilizing helical structures in polypeptides and oligomers [44]. A significant enhancement in CD intensity at 355 and 275 nm was observed for all SDOUs compared to MeOH, indicating conformational reorganization (Figure 8C). The spectral pattern suggests that **SC7** adopts a left‐handed helicity whereas the **RC7** and **SRC7** sequences exhibit right‐handed helicity, highlighting the role of chiral composition in determining overall secondary structure [45, 46].

These findings suggest that SDOUs can undergo polarity‐dependent folding, leading to tunable secondary structures. In non‐polar solvents like chloroform (CHCl₃) and tetrahydrofuran (THF), both bands follow expected positions corresponding to main‐chain chirality, suggesting stabilization of less ordered or extended conformers. However, in MeOH and TFE, an inversion in the dansyl‐associated bands indicates a solvent‐driven folding process where main‐chain orientation alters the dansyl moiety's positioning, thereby influencing helicity. **SRC7**, which remained nearly silent in CHCl_3_ and THF, displayed a spectral pattern in polar solvents resembling **RC7**, implying that its R‐configured segments near the dansyl group dominate overall chiral behavior, favoring right‐handed helicity. While these results provide preliminary insights into solvent‐driven conformational dynamics, further experimental and theoretical investigations are needed to elucidate the precise folding mechanisms and their impact on CD spectral behavior.

## 3 Conclusions

In this study, we developed an efficient, Fmoc‐assisted solution‐phase synthetic strategy for stereo‐regulated, sequence‐defined oligourethanes (SDOUs), overcoming significant challenges in scalability, yield, and time efficiency. The two‐step, one‐pot deprotection‐coupling methodology facilitated rapid synthesis without intermediate purification, enabling the production of oligourethanes with precise sequence control and high stereochemical fidelity. We synthesized three chiral sequences with distinct chirality patterns to explore the impact of monomer sequence and chirality on material properties. Thermal analysis revealed the significant role of stereochemistry in governing the glass transition, crystallization, and melting behaviors of the oligomers, correlating the intricate relationship between sequence chirality and thermal properties. Furthermore, the modularity of SDOUs was demonstrated through post‐synthetic modification with dansyl chloride, presenting their versatility for application‐specific modifications, elongation of the sequence, and functional tailoring of properties. Photophysical studies of dansyl‐functionalized SDOUs demonstrated their solvent‐dependent solvatochromism and distinct aggregation patterns, highlighting potential applications in bioimaging and sensing. Circular dichroism analysis provided evidence of the enantiomeric configurations and conformational stability of the oligomers. The fast and scalable protocol presented here provides a robust platform for the synthesis of sequence‐defined oligomers and can be utilized for polymer material development. The present study is an initial demonstration of perfect sequencing till 7‐mer, while the future goal focuses on the elongation of the sequence by strategic modifications of monomers. In this context, the work establishes a foundation for future exploration of stereo‐regulated polymers in advanced material applications, bridging the gap between natural precision and synthetic innovation.

## Conflicts of Interest

The authors declare no conflicts of interest.

## Supporting information




**Supporting File 1**: marc202500378‐sup‐0001‐SuppMat.docx.

## Data Availability

The data that support the findings of this study are available in the supplementary material of this article.

## References

[1] J. F. Lutz , "Defining the Field of Sequence‐Controlled Polymers,"Macromolecular Rapid Communications 38 (2017): 1–12. 10.1002/marc.201700582 29160615

[2] Z. Deng , Q. Shi , J. Tan , J. Hu , and S. Liu , “Sequence‐Defined Synthetic Polymers for New‐Generation Functional Biomaterials,” ACS Materials Letters 3, no. 9 (2021): 1339–1356.

[3] J. F. Lutz , J. M. Lehn , E. W. Meijer , and K. Matyjaszewski , “From Precision Polymers to Complex Materials and Systems,” Nature Reviews Materials 1 (2016): 16024.

[4] M. A. R. Meier and C. Barner‐Kowollik , “A New Class of Materials: Sequence‐Defined Macromolecules and Their Emerging Applications,” Advanced Materials 31, no. 26 (2019): 1806027. 10.1002/adma.201806027 30600565

[5] A. J. Destefano , R. A. Segalman , and E. C. Davidson “Where Biology and Traditional Polymers Meet: The Potential of Associating Sequence‐Defined Polymers for Materials Science,” JACS Au 1 (2021): 1556–1571. 34723259 10.1021/jacsau.1c00297 PMC8549048

[6] C. Yang , K. B. Wu , Y. Deng , J. Yuan , and J. Niu , “Geared toward Applications: A Perspective on Functional Sequence‐Controlled Polymers,” ACS Macro Letters 10 (2021): 243–257. 34336395 10.1021/acsmacrolett.0c00855 PMC8320758

[7] A. C. Deacy , G. L. Gregory , G. S. Sulley , T. T. D. Chen , and C. K. Williams , “Sequence Control from Mixtures: Switchable Polymerization Catalysis and Future Materials Applications,” Journal of the American Chemical Society 143 (2021): 10021–10040. 34190553 10.1021/jacs.1c03250 PMC8297863

[8] H. Ji , B. Wang , L. Pan , and Y. Li , “One‐Step Access to Sequence‐Controlled Block Copolymers by Self‐Switchable Organocatalytic Multicomponent Polymerization,” Angewandte Chemie 130 (2018): 17130–17134. 10.1002/anie.201810083 30417592

[9] Q. Shi , H. Yin , R. Song , et al., “Digital Micelles of Encoded Polymeric Amphiphiles for Direct Sequence Reading and Ex Vivo Label‐Free Quantification,” Nature Chemistry 15 (2022): 257–270. 10.1038/s41557-022-01076-y 36329179

[10] S. Martens , A. Landuyt , P. Espeel , B. Devreese , P. Dawyndt , and F. D.u Prez , “Multifunctional Sequence‐Defined Macromolecules for Chemical Data Storage,” Nature Communications 9 (2018): 4451. 10.1038/s41467-018-06926-3 PMC6203848 30367037

[11] T. Mondal , L. Charles , and J. F. Lutz , “Damage and Repair in Informational Poly( N ‐Substituted Urethane)s,” Angewandte Chemie, International Edition 59, no. 46 (2020): 20390–20393. 32779792 10.1002/anie.202008864

[12] K. Hakobyan , B. B. Noble , and J. Xu , “The Current Science of Sequence‐defined Macromolecules,” Progress in Polymer Science 147 (2023): 101754.

[13] E. M. Timmers , P. M. Fransen , J. R. Magana , H. M. Janssen , and I. K. Voets , “Micellization of Sequence‐Controlled Polyurethane Ionomers in Mixed Aqueous Solvents,” Macromolecules 54 (2021): 2376–2382. 33814615 10.1021/acs.macromol.0c02107 PMC8016144

[14] J. F. Lutz , “Sequence‐Controlled Polymerizations: The next Holy Grail in Polymer Science?,” Polymer Chemistry 1 (2010): 55–62.

[15] D. Karamessini , S. Poyer , L. Charles , and J. F. Lutz , “2D Sequence‐Coded Oligourethane Barcodes for Plastic Materials Labeling,” Macromolecular Rapid Communications 38 (2017): 1700426. 10.1002/marc.201700426 28833851

[16] M. Soete , J. Van Hoorde , and F. D.u Prez , “Discrete, Self‐Immolative N ‐Substituted Oligourethanes and Their Use as Molecular Tags,” Polymer Chemistry 13, no. 28 (2022): 4178–4185.

[17] J. Cen , M. Hou , J. Hu , and S. Liu “Advanced Synthesis, Structural Characterization, and Functional Applications of Precision Polymers,” Chemistry–A European Journal. 30, 56 (2024) 202401911. 10.1002/chem.202401911 39079912

[18] E. A. Hoff , G. X. De Hoe , C. M. Mulvaney , M. A. Hillmyer , and C. A. Alabi , “Thiol–Ene Networks from Sequence‐Defined Polyurethane Macromers,” Journal of the American Chemical Society 142, no. 14 (2020): 6729–6736. 32202773 10.1021/jacs.0c00759

[19] J. Li , M. Leclercq , M. Fossepré , et al., “Discrete Multifunctional Sequence‐Defined Oligomers with Controlled Chirality,” Polymer Chemistry 11, no. 24 (2020): 4040–4046.

[20] I. De Franceschi , C. Mertens , N. Badi , and F. D.u Prez , “Uniform Soluble Support for the Large‐Scale Synthesis of Sequence‐Defined Macromolecules,” Polymer Chemistry 13, no. 39 (2022): 5616–5624.

[21] P. Cwynar , P. Pasikowski , and R. Szweda , “One‐Pot Approach for Multi‐Step, Iterative Synthesis of Sequence‐Defined Oligocarbamates,” European Polymer Journal 182 (2023): 111706.

[22] C. Y. Cho , E. J. Moran , S. R. Cherry , et al., “An Unnatural Biopolymer,” Science 261, no. 5126 (1993): 1303–1305. 7689747 10.1126/science.7689747

[23] U. S. Gunay , B. E. Petit , D. Gigmes , and J. F. Lutz , “Chemoselective Synthesis of Uniform Sequence‐Coded Polyurethanes and Their Use as Molecular Tags,” Chemistry 1, no. 1 (2016): 114–126.

[24] Q. Shi , Z. Deng , M. Hou , X. Hu , and S. Liu , “Engineering Precise Sequence‐Defined Polymers for Advanced Functions,” Progress in Polymer Science 141 (2023): 101677.

[25] J. O. Holloway , K. S. Wetzel , S. Martens , F. Du Prez , and M. A. R. Meier , “Direct Comparison of Solution and Solid Phase Synthesis of Sequence‐Defined Macromolecules,” Polymer Chemistry 10, no. 28 (2019): 3859–3867.

[26] C. Mertens , M. Soete , M. L. Ślȩczkowski , et al., “Stereocontrolled, Multi‐Functional Sequence‐Defined Oligomers Through Automated Synthesis,” Polymer Chemistry 11, no. 26 (2020): 4271–4280.

[27] S. D. Dahlhauser , P. R. Escamilla , A. N. Vandewalle , et al., “Sequencing of Sequence‐Defined Oligourethanes via Controlled Self‐Immolation,” Journal of the American Chemical Society 142, no. 6 (2020): 2744–2749. 31986251 10.1021/jacs.9b12818 PMC8573737

[28] R. L. Kanasty , A. J. Vegas , L. M. Ceo , et al., “Sequence‐Defined Oligomers from Hydroxyproline Building Blocks for Parallel Synthesis Applications,” Angewandte Chemie 128, no. 33 (2016): 9681–9685. 10.1002/anie.201602748 PMC5245870 27365192

[29] Y. Song , C. Sun , C. Tian , et al., “Precisely Synthesized Segmented Polyurethanes toward Block Sequence‐Controlled Drug Delivery,” Chemical Science 13, no. 18 (2022): 5353–5362. 35655572 10.1039/d1sc06457f PMC9093123

[30] E. M. Timmers , P. M. Fransen , Á. G. Garciá , et al, “Co‐assembly of Precision Polyurethane Ionomers Reveals Role of and Interplay between Individual Components,” Polymer Chemistry 12, no. 19 (2021): 2891–2903. 34046093 10.1039/d1py00079a PMC8129887

[31] T. Mondal , V. Greff , B. É. Petit , L. Charles , and J. F. Lutz , “Efficient Protocol for the Synthesis of “N‐Coded” Oligo‐and Poly (N‐Substituted Urethanes).ACS Macro Letter,” ACS Macro Letters 8, no. 8 (2019): 1002–1005. 35619476 10.1021/acsmacrolett.9b00446

[32] J. A. Amalian , S. Poyer , B. E. Petit , et al., “Negative Mode MS/MS to Read Digital Information Encoded in Sequence‐Defined Oligo(Urethane)s: A Mechanistic Study,” International Journal of Mass Spectrometry. 421 (2017): 271–278.

[33] X. Wang , I. Huq , and T. M. Rana , “HIV‐1 TAR RNA Recognition by an Unnatural Biopolymer,” Journal of the American Chemical Society 119, no. 27 (1997): 6444–6445.

[34] I. De Franceschi , N. Badi , and F. E. Du Prez , “Telechelic Sequence‐defined Oligoamides: Their Step‐economical Synthesis, Depolymerization and Use in Polymer Networks,” Chemical Science 15, no. 8 (2024): 2805–2816. 38404375 10.1039/d3sc04820a PMC10882489

[35] R. Corradini , A. Dossena , R. Marchelli , et al., “A Modified Cyclodextrin with a Fully Encapsulated Dansyl Group: Self‐Inclusion in the Solid State and in Solution,” Chemistry—A European Journal 2 (1996): 373–381.

[36] N. M. H. Salem , S. Foro , and M. F. Iskander , “Metal Complexes Derived from Hydrazoneoxime Ligands VI. Synthesis, Characterization and Structures of Zinc(II) Complexes Derived from Aroylhydrazoneoximes,” Journal of Molecular Structure 1227 (2021): 129571.

[37] Y. Li , L. Chan , L. Tyer , R. T. Moody , C. M. Himel , and D. M. Hercules , “Solvent Effects on the Fluorescence of 1‐(dimethylamino)‐5‐naphthalenesulfonic Acid and Related Compounds,” Journal of the American Chemical Society 97 (1975): 3118–3126.

[38] L. Thurakkal , K. M. Mol , and M. R. Porel , “Dansyl‐triazole‐based Fluorescent Macrocycle for Selective Detection of Nitro‐antibiotic Drugs and Protein Interaction,” Chemical Communications 59, no. 48 (2023): 7399–7402. 37232580 10.1039/d3cc01769a

[39] X. Qin , X. Yang , L. Du , and M. Li , “Polarity‐Based Fluorescence Probes: Properties and Applications,” RSC Medicinal Chemistry 12, no. 11 (2021): 1826–1838. 34825183 10.1039/d1md00170a PMC8597426

[40] D. Sahoo , P. Bhattacharya , and S. Chakravorti , “Spectral Signature of 2‐[4‐(dimethylamino) styryl]‐1‐methylquinolinium Iodide: A Case of Negative Solvatochromism in Water,” The Journal of Physical Chemistry B. 115, no. 37 (2011): 10983‐10989. 21827188 10.1021/jp2046239

[41] E. K. Stacy , M. L. McCormick , K. C. Stevens , et al., “Aqueous Photoiniferter Polymerization of Acrylonitrile,” ACS Macro Letters 13 (2024): 1662–1669. 39560607 10.1021/acsmacrolett.4c00642 PMC11656713

[42] M. Vasilescu , E. Pincu , V. Meltzer , and G. Ionita , “Modulation of Dansyl Moiety Fluorescence in Systems Containing Cyclodextrins,” New Journal of Chemistry 36, no. 10 (2012): 2128–2134.

[43] A. Mukherjee , S. Barman , A. Ghosh , et al., “Stable Room Temperature Ferroelectricity in Hydrogen‐Bonded Supramolecular Assemblies of Ambipolar π‐Systems,” Chemical Science 13, no. 3 (2022): 781–788. 35173943 10.1039/d1sc04617a PMC8768847

[44] D. Roccatano , G. Colombo , M. Fioroni , and A. E. Mark , “Mechanism by Which 2,2,2‐trifluoroethanol/Water Mixtures Stabilize Secondary‐structure Formation in Peptides: A Molecular Dynamics Study,” Proceedings of the National Academy of Sciences 99, no. 19 (2002): 12179–12184. 10.1073/pnas.182199699 PMC129418 12196631

[45] T. Fujiwara , Y. Taniguchi , Y. Katsumoto , et al., “Induced Circular Dichroism in Chiral N‐Methyl Amides Possessing an Achiral Binaphthyl Chromophore and Its Application to Absolute Configuration Determination of Aliphatic Chiral Amines,” Tetrahedron: Asymmetry 23, no. 13 (2012): 981–991.

[46] G. Gottarelli , S. Lena , S. Masiero , S. Pieraccini , and G. P. Spada , “The Use of Circular Dichroism Spectroscopy for Studying the Chiral Molecular Self‐Assembly: An Overview,” Chirality 20 (2008): 471–485. 17918751 10.1002/chir.20459

