# Supplementary material for: Efficient Solution‐Phase Synthesis of Sequence‐Defined Oligourethanes with Precise Chirality Control
Source: Macromol Rapid Commun. 2025 Jun 22;46(18):e00378. doi: 10.1002/marc.202500378 (PMC12447683; doi:10.1002/marc.202500378)
Supplement: Supplementary file 1 — Supporting File 1: marc202500378‐sup‐0001‐SuppMat.docx. [file MARC-46-e00378-s001.docx]

**Supporting Information**

**Efficient Solution-Phase Synthesis of Sequence-Defined Oligourethanes with Precise Chirality Control**

**Anuj Sharma,^[a]^* Tapendu Samanta,^[a]^* Joanna Cybińska^[a,b]^**

^[a]^Łukasiewicz Research Network - PORT Polish Center for Technology Development, Stabłowicka 147, 54-066, Wrocław, Poland

^[b]^Department of Chemistry, University of Wrocław, F. Joliot-Curie 14, 50-300, Wrocław, Poland

*****  [Corresponding authors: anuj.sharma@port.lukasiewicz.gov.pl](mailto:%20Corresponding%20authors:%20anuj.sharma@port.lukasiewicz.gov.pl); [tapendu.samanta@port.lukasiewicz.gov.pl](mailto:tapendu.samanta@port.lukasiewicz.gov.pl)

1. Materials and Methods

1.1 Materials

(S)-(+)-2-Amino-1-propanol (Ds, 99.9%, Ambeed), (2R)-(-)-2-Aminopropan-1-ol (Dr, 99.9%, Ambeed), (2S)-(+)-1-Aminopropan-2-ol (Cs, 98%, Ambeed), (R)-(-)-1-Aminopropan-2-ol (Cr, 95%, Fluorochem), L-Valinol (Vs, 98%, Angene), D-valinol (Vr, 98%, Angene), Benzylamine (99%, Sigma Aldrich), N,N'-Disuccinimidyl carbonate (DSC, 98%, Angene), 9-fluorenemethanol (Fmoc-OH, 98%, Ambeed), Sodium hydrogen carbonate (99.5%, Chempur), Piperidine (99%, Sigma Aldrich), Pyridine (99.8%, anhydrous, Sigma-Aldrich), Acetonitrile (99.8%, anhydrous, Sigma-Aldrich), N,N-Dimethylformamide (99.8%, anhydrous, Sigma-Aldrich), Dichloromethane (DCM, >99.5%, POCH-Avantor), Methanol (99.9%,Chemsolute), Ethyl acetate (99.5%,Chemsolute), Acetone (≥99.5%,Fisher Scientific), Sodium chloride (≥99.5%, Sigma-Aldrich), Sodium sulfate (≥99.0%, anhydrous, Sigma-Aldrich) were used as received from the suppliers.

**1.2 Methods**

**1.2.1 Nuclear Magnetic Resonance (NMR)**

^1^H NMR (500 MHz), spectra were recorded in deuterated chloroform (99.8 atom % D, Deutero) or deuterated dimethyl sulfoxide (99.8 atom% D, Sigma Aldrich) using Avance III HD 500 MHZ NMR Spectrometer (BRUKER) equipped with probes: BBI and BBO. The recoded spectra were assessed using MestReNova software.

**1.2.2 Reverse Phase High Performance Liquid Chromatography (RP-HPLC)**

The reverse phase high performance liquid chromatography (RP-HPLC) was used for examining of the oligomer synthesis. Analyses were performed using Thermo Scientific Dionex UltiMate 3000 with vacuum degasser SRD-3200, two-component quaternary pump, autosampler UltiMate™ WPS-3000SL/TSL, column thermostat TCC-3000SD, and diode array detector DAD-3000. The system was equipped with reverse phase Hypersil Gold C18 (150x4.6mm 5u Hypersil GOLD) column attached with guard. The chromatograms were recorded in λ=190-400 nm range. Experimental conditions: phase A: 10% ACN (HPLC grade, ≥99.9%, Chemsolute) in water (MiliQ), phase B: 10% ACN (HPLC grade, ≥99.9%, Chemsolute); gradient elution: 0-0.667 min 10% B, 0.667-5.0 min 10-100% B, 5.0-5.667 min 100% B, 5.667-6.0 min 100-10% B; flow rate: 1.5 mL·min^-1^; T=25 ^o^C. The chromatograms were analyzed at 220 nm and 260 nm using Chromeleon software.

**1.2.3 High resolution - Mass Spectrometry (HR-MS)**

High-resolution mass spectrometry (HRMS) measurements were performed using a **Bruker Daltonics maXis impact Q-ToF spectrometer** (Bruker Daltonics, Germany) equipped with an **electrospray ionization (ESI) source.** The oligourethane samples were dissolved in **DMF at a concentration of 1 mg/mL** and further diluted to a final concentration of **50 µg/mL** before analysis. The spectrometer operated in **MS mode**, maintaining an **ion energy of 5.0 eV.** The **ESI source parameters** were set as follows: **end plate offset 0.5 kV, capillary voltage 4 kV, nebulizing gas 2 bar, drying gas 8.0 L/min, and drying gas temperature 220°C**. Data were processed using **Data Analysis 4.1 software**, and molecular formulas were assigned using the **SmartFormula algorithm**.

**1.2.4 Size-exclusion chromatography (SEC)**

The SEC setup consist of Agilent system equipped with RI detectors mixed PLGel E 3 μm, 300 x 7.5 mm column. For analysis THF (HPLC grade, BHT stabilised, Thermo Fisher) was used as mobile phase (0.5 mL/min flow rate) depending on the solubility of oligomers.

**1.2.5 Thermogravimetric Analysis (TGA)**

Thermogravimetric analysis (TGA) was performed using a Mettler Toledo TGA 2 Thermogravimetric Analyzer with a large furnace (LF). Approximately 5 mg of each sample was analyzed in a nitrogen atmosphere (30 mL/min) over a temperature range of 25 °C to 600 °C with a heating rate of 10 K/min.

**1.2.6 Differential Scanning Calorimetry (DSC)**

Differential scanning calorimetry (DSC) measurements were conducted using a Mettler Toledo Differential Scanning Calorimeter under a nitrogen atmosphere (50 mL/min) within a temperature range of -50 °C to 170 °C. Each sample (~3 mg) was sealed in an aluminum pan, and indium was used as a reference substance. The experiments consisted of two cycles of heating-cooling-heating, performed at a rate of 10 K/min. Prior to the measurements, the samples were preheated in an oil bath at 100 °C under vacuum for 5–10 minutes to remove solvent traces. Glass transition temperatures (Tg) were determined from the inflection point of the second heating curve, and melting temperatures (Tm) were determined as the minimum of the first endothermic peak and crystallization temperature (Tc) were determine as the minimum of the first exothermic peak.

**1.2.7 Circular dichroism (CD)**

Circular dichroism (CD) spectra of the oligourethanes were recorded using a Jasco J-815 spectropolarimeter. The measurements were performed at 25 °C with the following parameters: scanning speed of 100 nm/min, bandwidth of 1.00 nm, digital integration time (D.I.T.) of 1 second, and three accumulations per sample. A quartz cuvette with a path length of 10 mm was used for all measurements. The oligourethane samples were prepared by weighing 1mg in 2mL of the desired solvent.

**1.2.8 UV-Vis Spectroscopy**

UV-Vis absorption spectra of the oligourethanes were recorded using a Thermo Scientific Evolution 201 UV-Visible Spectrophotometer. Solutions were prepared at a concentration of 10 μM. All measurements were carried out in a quartz cuvette with a 10 mm path length at room temperature, and the spectra were recorded in the wavelength range 240 nm to 450 nm.

**1.2.9 Photoluminescence (PL) Spectroscopy**

Fluorescence spectra were measured using a SCINCO Fluorescence Spectrometer FluoroMate FS-2. The experimental processes were controlled using FluoroMaster Plus software. Samples for fluorescence measurements were prepared in desired solvent at a concentration of 10 μM in a quartz cuvette with a 10 × 10 mm light path. Spectra were recorded at an excitation wavelength of 340 nm, with the emission spectra collected in the range of 350–700 nm. Measurements were conducted at room temperature.

**1.2.10 Dynamic Light Scattering (DLS)**

Dynamic light scattering (DLS) measurements were carried out using a **Malvern Zetasizer Nano ZS** (Malvern Panalytical, UK). Samples were prepared at a concentration of 10 μM in the desired water-THF mixture and loaded into a glass cuvette for measurement. The measurements were performed at room temperature, and the data were analyzed using the Zetasizer software provided with the instrument to determine the hydrodynamic size and polydispersity index (PDI) of the samples.

1. Experimental procedures

2.1 General synthetic procedure for modified chiral monomer:

A mixture of (9H-fluoren-9-yl)methyl (2,5-dioxopyrrolidin-1-yl) carbonate (Fmoc-OSu) (1 equivalent), sodium hydrogen carbonate (1 equivalent) and acetonitrile:water (3:2) was taken in a round bottom flask and added chiral amino alcohol (1.1 equivalent). The reaction mixture was stirred for 2h at room temperature and completion of reaction was confirmed by reverse-phase HPLC. The excess acetonitrile was reduced under pressure and organic compound was extracted with ethyl acetate and water, three times. The organic solvent was evaporated under reduced pressure to obtain the white solid powder which was used in the next step without further purification. The resulting Fmoc-protected compound (Fmoc-X-OH) (1 equivalent) and N,N'-disuccinimidyl carbonate (1.5 equivalent) was taken in a round bottom flask followed by addition of anhydrous acetonotrile (0.2 M) and pyridine (1.2 equivalent) under inert atmosphere. The reaction mixture was stirred for 12h at room temperature. The progress of the reaction was monitored by reverse-phase HPLC and upon completion of reaction, the acetonitrile was reduced under pressure. The organic compound was added with ethyl acetate and washed with water and brine solution for three times. The organic solvent was dried over rotary evaporator to obtain the product (Fmoc-X-OSu) as white powder. The obtained monomers was used without further purification.

Figure S1. General synthetic scheme for monomers.

**2.2 General synthetic procedure for sequence-defined oligourethanes**

Dissolve benzylamine (1eq) in dimethylformamide (DMF) as the nucleophilic starting material, and add the desired Fmoc-protected monomer (1eq). Stir the reaction mixture for 15 minutes at room temperature, confirming completion of the coupling reaction via reverse-phase HPLC. For deprotection, add 20% (v/v) piperidine in DMF, stir for 15 minutes. Dry the mixture using a rotary evaporator, co-evaporate with ethyl acetate to remove residual piperidine, and proceed directly with the addition of the next Fmoc-protected monomer (1eq) without purification. Repeat the coupling and deprotection steps iteratively until the desired oligomer sequence is synthesized. After each step, perform liquid-liquid extraction with water and ethyl acetate to remove unreacted reagents and byproducts such as N-hydroxysuccinimide, Fmoc-piperidine adduct and dibenzofulvene, and precipitate the product by adding the mixture to hexanes (100mL). The iterative process is continued until the target oligomer is achieved, with progress monitored at each step using reverse-phase HPLC. This one-pot deprotection-coupling method consistently achieves high yields of 90–99% per step, with overall yields of 67% (**SC6**), 70% (**RC6**), and 72% (**SRC6**), and results in highly pure oligomers, as confirmed by HPLC, GPC, and NMR.

**Figure S2.** Synthesis scheme of **SC6** sequence.

**Figure S3.** Synthesis scheme of **RC6** sequence.

**Figure S4.** Synthesis scheme of **SRC6** sequence.

**Table S1.** Molecular Weight, step yields, overall yield and scale of synthesized chirality and sequence-defined oligourethanes (**SC6**).

| **Steps** | **Sequence** | **MW** | **Yield (%)** | **Overall yield (%)** |
| --- | --- | --- | --- | --- |
| 1 | **SC1** | 430.504 | 99 |  |
| 2 | **SC2** | 531.609 | 92 |  |
| 3 | **SC3** | 660.708 | 91 | 67 |
| 4 | **SC4** | 761.873 | 93 |  |
| 5 | **SC5** | 862.978 | 95 |  |
| 6 | **SC6** | 992.137 | 91 |  |

**Table S2.** Molecular Weight, step yields, overall yield and scale of synthesized chirality and sequence-defined oligourethanes (**RC6**).

| **Steps** | **Sequence** | **MW** | **Yield (%)** | **Overall yield (%)** |
| --- | --- | --- | --- | --- |
| 1 | **RC1** | 430.504 | 86 |  |
| 2 | **RC2** | 531.609 | 95 |  |
| 3 | **RC3** | 660.708 | 96 | 72 |
| 4 | **RC4** | 761.873 | 96 |  |
| 5 | **RC5** | 862.978 | 98 |  |
| 6 | **RC6** | 992.137 | 98 |  |

**Table S3.** Molecular Weight, step yields, overall yield and scale of synthesized chirality and sequence-defined oligourethanes (**SRC6**).

| **Steps** | **Sequence** | **MW** | **Yield (%)** | **Overall yield (%)** |
| --- | --- | --- | --- | --- |
| 1 | **SRC1** | 430.504 | 96 |  |
| 2 | **SRC2** | 531.61 | 92 |  |
| 3 | **SRC3** | 632.71 | 96 | 70 |
| 4 | **SRC4** | 733.82 | 94 |  |
| 5 | **SRC5** | 862.98 | 98 |  |
| 6 | **SRC6** | 992.14 | 90 |  |

**Figure S5.** HPLC spectra of sequence synthesis from monomer (**SC1**) to hexamer (**SC6**).

**Figure S6.** HPLC spectra of sequence synthesis from monomer (**RC1**) to hexamer (**RC6**).

**Figure S7.** HPLC spectra of sequence synthesis from monomer (**SRC1**) to hexamer (**SRC6**).

**(9H-fluoren-9-yl)methyl (S)-(1-hydroxypropan-2-yl)carbamate (Fmoc-Ds-OH)**

^1^H NMR (500 MHz, CDCl_3_) δ 7.77 (d, *J* = 7.5 Hz, 2H), 7.59 (d, *J* = 7.5 Hz, 2H), 7.41 (t, *J* = 7.4 Hz, 2H), 7.32 (t, *J* = 7.4 Hz, 2H), 4.85 (s, 1H), 4.43 (s, 2H), 4.22 (t, *J* = 6.5 Hz, 1H), 3.84 (s, 1H), 3.67 (d, *J* = 8.9 Hz, 1H), 3.54 (s, 1H), 1.17 (s, 3H).

^13^C NMR (126 MHz, CDCl_3_) δ 143.93 (s), 141.37 (s), 127.71 (s), 127.08 (s), 124.99 (s), 119.99 (s), 77.28 (s), 77.02 (s), 76.77 (s), 66.68 (s), 49.00 (s), 47.32 (s).

**Figure S8.** ^1^H NMR spectrum of **Fmoc-Ds-OH** recorded in CDCl_3_.

**Figure S9.** ^13^C NMR spectrum of **Fmoc-Ds-OH** recorded in CDCl_3_.

**(9H-fluoren-9-yl)methyl (R)-(1-hydroxypropan-2-yl)carbamate (Fmoc-Dr-OH)**

^1^H NMR (500 MHz, CDCl_3_) δ 7.77 (d, *J* = 7.6 Hz, 2H), 7.59 (d, *J* = 7.5 Hz, 2H), 7.40 (t, *J* = 7.4 Hz, 2H), 7.32 (td, *J* = 7.4, 0.9 Hz, 2H), 4.89 (s, 1H), 4.44 (t, *J* = 8.5 Hz, 2H), 4.21 (t, *J* = 6.6 Hz, 1H), 3.93 – 3.76 (m, 1H), 3.65 (s, 1H), 3.58 – 3.47 (m, 1H), 2.03 (s, 1H), 1.17 (d, *J* = 4.7 Hz, 3H).

^13^C NMR (126 MHz, CDCl_3_) δ 143.90, 143.87, 141.33, 127.68, 127.04, 127.03, 125.00, 124.96, 119.95, 77.25, 77.00, 76.75, 67.07, 66.64, 48.95, 47.28, 17.25.

**Figure S10.** ^1^H NMR spectrum of **Fmoc-Dr-OH** recorded in CDCl_3_.

**Figure S11.** ^13^C NMR spectrum of **Fmoc-Dr-OH** recorded in CDCl_3_.

**(9H-fluoren-9-yl)methyl (S)-(2-hydroxypropyl)carbamate (Fmoc-Cs-OH)**

^1^H NMR (500 MHz, CDCl_3_) δ 7.76 (d, *J* = 7.6 Hz, 2H), 7.59 (d, *J* = 7.4 Hz, 2H), 7.40 (t, *J* = 7.4 Hz, 2H), 7.31 (td, *J* = 7.5, 1.1 Hz, 2H), 5.19 (s, 1H), 4.44 (t, *J* = 9.6 Hz, 2H), 4.22 (t, *J* = 6.7 Hz, 1H), 3.91 (s, 1H), 3.34 (d, *J* = 13.1 Hz, 1H), 3.06 (s, 1H), 2.03 (s, 1H), 1.18 (d, *J* = 5.7 Hz, 3H).

^13^C NMR (126 MHz, CDCl_3_) δ 143.87, 141.33, 127.68, 127.03, 124.98, 119.95, 77.25, 77.00, 76.75, 67.39, 66.73, 48.22, 47.25, 20.67.

**Figure S12.** ^1^H NMR spectrum of **Fmoc-Cs-OH** recorded in CDCl_3_.

**Figure S13.** ^13^C NMR spectrum of **Fmoc-Cs-OH** recorded in CDCl_3_.

**(9H-fluoren-9-yl)methyl (R)-(2-hydroxypropyl)carbamate (Fmoc-Cr-OH)**

^1^H NMR (500 MHz, CDCl3) δ 7.71 (d, *J* = 7.5 Hz, 2H), 7.53 (d, *J* = 7.4 Hz, 2H), 7.34 (t, *J* = 7.4 Hz, 2H), 7.25 (t, *J* = 8.0 Hz, 2H), 5.11 (s, 1H), 4.37 (d, *J* = 6.5 Hz, 2H), 4.16 (t, *J* = 6.7 Hz, 1H), 3.85 (s, 1H), 3.28 (d, *J* = 12.8 Hz, 1H), 3.00 (s, 1H), 1.92 (s, 1H), 1.13 (d, *J* = 5.8 Hz, 3H).

^13^C NMR (126 MHz, CDCl_3_) δ 143.92 (s), 141.37 (s), 127.72 (s), 127.07 (s), 125.02 (s), 119.99 (s), 67.46 (s), 66.77 (s), 48.26 (s), 47.29 (s), 20.72 (s).

**Figure S14.** ^1^H NMR spectrum of **Fmoc-Cr-OH** recorded in CDCl_3_.

**Figure S15.** ^13^C NMR spectrum of **Fmoc-Cr-OH** recorded in CDCl_3_.

**(9H-fluoren-9-yl)methyl (S)-(1-hydroxy-3-methylbutan-2-yl)carbamate (Fmoc-Vs-OH)**

^1^H NMR (500 MHz, CDCl_3_) δ 7.69 (d, *J* = 7.5 Hz, 2H), 7.52 (d, *J* = 7.5 Hz, 2H), 7.32 (t, *J* = 7.5 Hz, 2H), 7.24 (t, *J* = 8.0 Hz, 2H), 4.79 (d, *J* = 6.6 Hz, 1H), 4.45 – 4.30 (m, 2H), 4.15 (t, *J* = 6.6 Hz, 1H), 3.71 – 3.47 (m, 2H), 3.40 (s, 1H), 1.85 (s, 1H), 1.82 – 1.72 (m, 1H), 0.86 (dt, *J* = 21.7, 10.8 Hz, 6H).

^13^C NMR (126 MHz, CDCl_3_) δ 143.94 (d, *J* = 3.9 Hz), 141.37 (s), 127.70 (s), 127.08 (s), 125.01 (s), 119.99 (s), 66.61 (s), 63.80 (s), 58.62 (s), 47.39 (s), 29.24 (s), 19.51 (s), 18.67 (s).

**Figure S16.** ^1^H NMR spectrum of **Fmoc-Vs-OH** recorded in CDCl_3_.

**Figure S17.** ^13^C NMR spectrum of **Fmoc-Vs-OH** recorded in CDCl_3_.

**(9H-fluoren-9-yl)methyl (S)-(1-hydroxy-3-methylbutan-2-yl)carbamate (Fmoc-Vr-OH)**

^1^H NMR (500 MHz, CDCl_3_) δ 7.71 (d, *J* = 7.5 Hz, 2H), 7.54 (d, *J* = 7.5 Hz, 2H), 7.34 (t, *J* = 7.5 Hz, 2H), 7.26 (t, *J* = 7.4 Hz, 2H), 4.81 (s, 1H), 4.48 – 4.31 (m, 2H), 4.16 (t, *J* = 6.6 Hz, 1H), 3.61 (dt, *J* = 16.3, 8.4 Hz, 2H), 3.41 (s, 1H), 1.89 (s, 1H), 1.85 – 1.77 (m, 1H), 0.86 (dt, *J* = 47.7, 23.8 Hz, 6H).

^13^C NMR (126 MHz, CDCl_3_) δ 157.07 (s), 143.94 (d, *J* = 3.9 Hz), 141.39 (s), 127.70 (s), 127.08 (s), 125.01 (s), 119.99 (s), 77.29 (s), 77.04 (s), 76.78 (s), 66.62 (s), 63.80 (s), 58.62 (s), 47.39 (s), 29.24 (s), 19.50 (s), 18.66 (s).

**Figure S18.** ^1^H NMR spectrum of **Fmoc-Vr-OH** recorded in CDCl_3_.

**Figure S19.** ^13^C NMR spectrum of **Fmoc-Vr-OH** recorded in CDCl_3_.

**(9H-fluoren-9-yl)methyl (S)-(1-((((2,5-dioxopyrrolidin-1-yl)oxy)carbonyl)oxy)propan-2-yl)carbamate (Fmoc-Ds-OSu)**

^1^H NMR (500 MHz, CDCl_3_) δ 7.77 (d, J = 7.5 Hz, 2H), 7.60 (d, J = 7.0 Hz, 2H), 7.40 (t, J = 7.4 Hz, 2H), 7.33 (td, J = 7.4, 0.9 Hz, 2H), 4.88 (d, J = 6.7 Hz, 1H), 4.52 – 4.43 (m, 1H), 4.37 (dd, J = 21.8, 11.2 Hz, 3H), 4.23 (t, J = 6.7 Hz, 1H), 4.10 (s, 1H), 2.83 (s, 4H), 1.28 (d, J = 6.0 Hz, 3H).

^13^C NMR (126 MHz, CDCl_3_) δ 168.46 (s), 143.81 (s), 141.33 (s), 127.70 (s), 127.07 (d, J = 3.0 Hz), 125.07 (d, J = 12.0 Hz), 119.97 (s), 77.25 (s), 77.00 (s), 76.75 (s), 73.27 (s), 66.87 (s), 47.22 (s), 45.86 (s), 25.44 (s), 17.12 (s).

**Figure S20.** ^1^H NMR spectrum of **Fmoc-Ds-OSu** recorded in CDCl_3_.

**Figure S21.** ^13^C NMR spectrum of **Fmoc-Ds-OSu** recorded in CDCl_3_.

**(9H-fluoren-9-yl)methyl (R)-(1-((((2,5-dioxopyrrolidin-1-yl)oxy)carbonyl)oxy)propan-2-yl)carbamate (Fmoc-Dr-OSu)**

^1^H NMR (500 MHz, CDCl_3_) δ 7.77 (d, J = 7.5 Hz, 2H), 7.60 (d, J = 7.0 Hz, 2H), 7.40 (t, J = 7.4 Hz, 2H), 7.33 (td, J = 7.4, 0.9 Hz, 2H), 4.88 (d, J = 6.6 Hz, 1H), 4.54 – 4.43 (m, 1H), 4.37 (dd, J = 21.9, 11.4 Hz, 3H), 4.23 (t, J = 6.7 Hz, 1H), 4.10 (s, 1H), 2.83 (s, 4H), 1.28 (d, J = 6.2 Hz, 3H).

^13^C NMR (126 MHz, CDCl_3_) δ 168.47 (s), 155.53 (s), 151.58 (s), 143.86 (d, J = 11.6 Hz), 141.32 (s), 127.70 (s), 127.06 (s), 125.02 (s), 119.97 (s), 77.25 (s), 77.00 (s), 76.75 (s), 73.27 (s), 66.86 (s), 47.22 (s), 45.85 (s), 25.44 (s), 17.13 (s).

**Figure S22.** ^1^H NMR spectrum of **Fmoc-Dr-OSu** recorded in CDCl_3_.

**Figure S23.** ^13^C NMR spectrum of **Fmoc-Dr-OSu** recorded in CDCl_3_.

**(9H-fluoren-9-yl)methyl (S)-(2-((((2,5-dioxopyrrolidin-1-yl)oxy)carbonyl)oxy)propyl)carbamate (Fmoc-Cs-OSu)**

^1^H NMR (500 MHz, CDCl_3_) δ 7.77 (d, J = 7.5 Hz, 2H), 7.61 (d, J = 7.1 Hz, 2H), 7.40 (dd, J = 11.0, 3.9 Hz, 2H), 7.36 – 7.29 (m, 2H), 5.18 (t, J = 5.9 Hz, 1H), 5.00 (td, J = 6.6, 3.0 Hz, 1H), 4.42 (tt, J = 17.7, 9.0 Hz, 2H), 4.24 (t, J = 7.0 Hz, 1H), 3.55 (ddd, J = 14.8, 6.1, 3.0 Hz, 1H), 3.45 – 3.31 (m, 1H), 2.83 (s, 4H), 1.39 (d, J = 6.4 Hz, 3H).

^13^C NMR (126 MHz, CDCl_3_) δ 168.61 (s), 156.49 (s), 150.96 (s), 143.86 (d, J = 11.6 Hz), 141.28 (s), 127.67 (s), 127.05 (s), 125.10 (s), 119.93 (s), 78.91 (s), 77.25 (s), 77.00 (s), 76.75 (s), 67.04 (s), 47.18 (s), 45.04 (s), 25.44 (s), 16.82 (s).

**Figure S24.** ^1^H NMR spectrum of **Fmoc-Cs-OSu** recorded in CDCl_3_.

**Figure S25.** ^13^C NMR spectrum of **Fmoc-Cs-OSu** recorded in CDCl_3_.

**(9H-fluoren-9-yl)methyl (R)-(2-((((2,5-dioxopyrrolidin-1-yl)oxy)carbonyl)oxy)propyl)carbamate (Fmoc-Cr-OSu)**

^1^H NMR (500 MHz, CDCl_3_) δ 7.77 (d, J = 7.5 Hz, 2H), 7.61 (d, J = 7.2 Hz, 2H), 7.40 (t, J = 7.5 Hz, 2H), 7.32 (tt, J = 7.4, 1.2 Hz, 2H), 5.19 (t, J = 5.8 Hz, 1H), 5.00 (td, J = 6.6, 3.0 Hz, 1H), 4.41 (qd, J = 10.6, 7.3 Hz, 2H), 4.23 (t, J = 7.0 Hz, 1H), 3.55 (ddd, J = 14.8, 6.2, 3.0 Hz, 1H), 3.46 – 3.30 (m, 1H), 2.82 (s, 4H), 1.39 (d, J = 6.4 Hz, 3H).

^13^C NMR (126 MHz, CDCl_3_) δ: 168.63, 156.53, 151.01, 143.94, 143.85,141.33, 127.72, 127.10, 125.14, 119.98, 78.96, 67.10, 47.22, 45.09, 25.49, 16.87.

**Figure S26.** ^1^H NMR spectrum of **Fmoc-Cr-OSu** recorded in CDCl_3_.

**Figure S27.** ^13^C NMR spectrum of **Fmoc-Cr-OSu** recorded in CDCl_3_.

**(9H-fluoren-9-yl)methyl (S)-(1-((((2,5-dioxopyrrolidin-1-yl)oxy)carbonyl)oxy)-3-methylbutan-2-yl)carbamate (Fmoc-Vs-OSu)**

^1^H NMR (500 MHz, CDCl_3_) δ 7.71 (d, J = 7.7 Hz, 2H), 7.55 (dd, J = 7.1, 3.4 Hz, 2H), 7.34 (t, J = 7.5 Hz, 2H), 7.26 (td, J = 7.4, 1.1 Hz, 2H), 4.81 (d, J = 9.3 Hz, 1H), 4.58 – 4.28 (m, 4H), 4.19 (t, J = 6.8 Hz, 1H), 3.68 (tt, J = 14.9, 7.3 Hz, 1H), 2.76 (s, 4H), 1.90 – 1.75 (m, 1H), 0.92 (dd, J = 14.1, 6.7 Hz, 6H).

^13^C NMR (126 MHz, CDCl_3_) δ: 168.47, 156.10, 151.59, 143.86, 141.36, 127.70, 127.10, 125.16, 125.08, 119.98, 71.18, 66.86, 55.37, 47.33, 29.16, 25.46, 19.47, 18.67.

**Figure S28.** ^1^H NMR spectrum of **Fmoc-Vs-OSu** recorded in CDCl_3_.

**Figure S29.** ^13^C NMR spectrum of **Fmoc-Vs-OSu** recorded in CDCl_3_.

**(9H-fluoren-9-yl)methyl (R)-(1-((((2,5-dioxopyrrolidin-1-yl)oxy)carbonyl)oxy)-3-methylbutan-2-yl)carbamate (Fmoc-Vr-OSu)**

^1^H NMR (500 MHz, CDCl_3_) δ 7.71 (d, J = 7.7 Hz, 2H), 7.55 (dd, J = 7.1, 3.5 Hz, 2H), 7.34 (t, J = 7.5 Hz, 2H), 7.30 – 7.23 (m, 2H), 4.81 (d, J = 9.4 Hz, 1H), 4.55 – 4.24 (m, 4H), 4.19 (t, J = 6.8 Hz, 1H), 3.78 – 3.58 (m, 1H), 2.76 (s, 4H), 1.83 (dq, J = 13.4, 6.7 Hz, 1H), 0.92 (dd, J = 14.1, 6.7 Hz, 6H).

^13^C NMR (126 MHz, CDCl_3_) δ: 168.47, 156.10, 151.59, 143.86, 141.36, 127.70, 127.10, 125.16, 125.09, 119.98, 71.18, 66.86, 55.37, 47.33, 29.16, 25.46, 19.47, 18.67

**Figure S30.** ^1^H NMR spectrum of **Fmoc-Vr-OSu** recorded in CDCl_3_.

**Figure S31.** ^13^C NMR spectrum of **Fmoc-Vr-OSu** recorded in CDCl_3_.

**(9H-fluoren-9-yl)methyl (S)-(1-((benzylcarbamoyl)oxy)propan-2-yl)carbamate (SC1)**

^1^H NMR (500 MHz, DMSO) δ 7.89 (d, J = 7.5 Hz, 2H), 7.69 (t, J = 5.9 Hz, 3H), 7.41 (t, J = 7.4 Hz, 2H), 7.27 (dtd, J = 22.8, 15.2, 7.3 Hz, 8H), 4.36 – 4.11 (m, 5H), 3.88 (d, J = 6.1 Hz, 2H), 3.73 (dt, J = 13.5, 6.7 Hz, 1H), 1.05 (d, J = 6.5 Hz, 3H).

^13^C NMR (126 MHz, DMSO) δ 155.52, 143.89, 140.72, 128.19, 127.58, 127.00, 126.70, 125.13, 120.09, 39.35, 39.19, 39.02.

**Figure S32.** ^1^H NMR spectrum of **SC1** recorded in DMSO-d6.

**Figure S33.** ^13^C NMR spectrum of **SC1** recorded in DMSO-d6.

**(S)-1-((((9H-fluoren-9-yl)methoxy)carbonyl)amino)propan-2-yl((S)-1-((benzylcarbamoyl)oxy)propan-2-yl)carbamate (SC2)**

^1^H NMR (500 MHz, DMSO) δ 7.89 (d, J = 7.5 Hz, 2H), 7.68 (t, J = 7.3 Hz, 3H), 7.40 (q, J = 7.7 Hz, 3H), 7.31 (dd, J = 17.0, 7.6 Hz, 4H), 7.23 (dd, J = 14.2, 7.1 Hz, 3H), 6.96 (d, J = 7.8 Hz, 1H), 4.68 (dd, J = 12.2, 6.1 Hz, 1H), 4.32 – 4.14 (m, 5H), 3.89 – 3.64 (m, 3H), 3.18 – 3.03 (m, 2H), 1.05 (dd, J = 40.8, 6.2 Hz, 6H).

**Figure S34.** ^1^H NMR spectrum of **SC2** recorded in DMSO-d6.

**(9H-fluoren-9-yl)methyl ((6S,10S,16S)-6,10,17-trimethyl-3,8,13-trioxo-1-phenyl-4,9,14-trioxa-2,7,12-triazaoctadecan-16-yl)carbamate (SC3)**

^1^H NMR (500 MHz, DMSO) δ 7.89 (d, J = 7.5 Hz, 2H), 7.74 – 7.61 (m, 3H), 7.41 (t, J = 7.4 Hz, 2H), 7.32 (ddd, J = 16.9, 10.0, 5.3 Hz, 4H), 7.26 – 7.14 (m, 5H), 6.95 (d, J = 7.5 Hz, 1H), 4.65 (dd, J = 12.3, 6.1 Hz, 1H), 4.34 (t, J = 11.3 Hz, 1H), 4.19 (dd, J = 20.4, 6.7 Hz, 4H), 4.04 (dd, J = 10.8, 4.6 Hz, 1H), 3.85 (d, J = 4.1 Hz, 3H), 3.71 (dt, J = 13.6, 6.6 Hz, 1H), 3.52 (t, J = 13.8 Hz, 1H), 3.14 – 2.98 (m, 2H), 1.74 (dd, J = 13.0, 6.6 Hz, 1H), 1.05 (dd, J = 30.7, 6.3 Hz, 6H), 0.84 (dd, J = 9.5, 7.1 Hz, 6H).

**Figure S35.** ^1^H NMR spectrum of **SC3** recorded in DMSO-d6.

**(5S,10S)-1-(9H-fluoren-9-yl)-10-isopropyl-5-methyl-3,8-dioxo-2,7-dioxa-4,9-diazaundecan-11-yl ((6S,10S)-6,10-dimethyl-3,8-dioxo-1-phenyl-4,9-dioxa-2,7-diazaundecan-11-yl)carbamate (SC4)**

^1^H NMR (500 MHz, DMSO) δ 7.89 (d, J = 7.5 Hz, 2H), 7.68 (t, J = 8.6 Hz, 3H), 7.41 (t, J = 7.4 Hz, 2H), 7.32 (ddd, J = 14.7, 9.5, 4.2 Hz, 4H), 7.22 (dd, J = 14.7, 7.1 Hz, 4H), 7.16 (s, 1H), 7.03 (d, J = 9.0 Hz, 1H), 6.95 (d, J = 7.4 Hz, 1H), 4.65 (dd, J = 12.3, 6.1 Hz, 1H), 4.36 – 4.12 (m, 5H), 4.01 (dd, J = 10.9, 4.5 Hz, 1H), 3.94 – 3.67 (m, 7H), 3.50 (d, J = 13.8 Hz, 1H), 3.06 (d, J = 5.1 Hz, 2H), 1.71 (d, J = 6.3 Hz, 1H), 1.14 – 0.94 (m, 9H), 0.82 (dd, J = 11.7, 6.7 Hz, 6H).

**Figure S36.** ^1^H NMR spectrum **SC4** recorded in DMSO-d6.

**(6S,10S,15S)-1-(9H-fluoren-9-yl)-15-isopropyl-6,10-dimethyl-3,8,13-trioxo-2,7,12-trioxa-4,9,14-triazahexadecan-16-yl ((6S,10S)-6,10-dimethyl-3,8-dioxo-1-phenyl-4,9-dioxa-2,7-diazaundecan-11-yl)carbamate (SC5)**

^1^H NMR (500 MHz, DMSO) δ 7.89 (d, J = 7.5 Hz, 2H), 7.68 (d, J = 7.7 Hz, 3H), 7.40 (dd, J = 13.2, 5.8 Hz, 3H), 7.31 (dd, J = 17.4, 7.8 Hz, 4H), 7.23 (dd, J = 14.3, 7.1 Hz, 3H), 7.16 (s, 1H), 7.02 (d, J = 8.8 Hz, 1H), 6.94 (s, 2H), 4.66 (dt, J = 12.5, 6.1 Hz, 2H), 4.33 – 4.14 (m, 5H), 4.07 – 3.95 (m, 1H), 3.93 – 3.78 (m, 4H), 3.70 (dt, J = 12.8, 7.0 Hz, 3H), 3.49 (s, 1H), 3.07 (dd, J = 15.8, 5.7 Hz, 4H), 1.72 (d, J = 6.0 Hz, 1H), 1.14 – 0.95 (m, 12H), 0.83 (dd, J = 11.5, 6.8 Hz, 6H).

**Figure S37.** ^1^H NMR spectrum of **SC5** recorded in DMSO-d6.

**(5S,11S,15S,20S)-1-(9H-fluoren-9-yl)-5,20-diisopropyl-11,15-dimethyl-3,8,13,18-tetraoxo-2,7,12,17-tetraoxa-4,9,14,19-tetraazahenicosan-21-yl ((6S,10S)-6,10-dimethyl-3,8-dioxo-1-phenyl-4,9-dioxa-2,7-diazaundecan-11-yl)carbamate (SC6)**

^1^H NMR (500 MHz, DMSO) δ 7.89 (d, J = 7.5 Hz, 2H), 7.77 – 7.60 (m, 3H), 7.41 (t, J = 7.4 Hz, 2H), 7.36 – 7.27 (m, 4H), 7.27 – 7.13 (m, 5H), 7.02 (d, J = 8.6 Hz, 1H), 6.94 (s, 2H), 4.64 (dt, J = 11.7, 5.9 Hz, 2H), 4.34 (t, J = 11.3 Hz, 1H), 4.19 (dd, J = 19.1, 6.7 Hz, 4H), 4.02 (dd, J = 11.5, 7.1 Hz, 2H), 3.95 – 3.78 (m, 5H), 3.72 (dd, J = 13.3, 8.3 Hz, 3H), 3.50 (s, 2H), 3.06 (d, J = 5.3 Hz, 4H), 1.83 – 1.63 (m, 2H), 1.16 – 0.92 (m, 12H), 0.91 – 0.65 (m, 12H).

^13^C NMR (126 MHz, DMSO) δ 156.39, 156.33, 156.20, 156.14, 155.17, 143.98, 143.81, 140.73, 139.78, 128.21, 127.60, 127.02, 126.97, 126.72, 125.20, 120.10, 85.73, 68.84, 66.42, 66.20, 65.27, 64.10, 55.15, 46.79, 45.74, 44.93, 43.73, 30.94, 29.29, 22.05, 19.18, 17.85, 17.34, 13.95.

HRMS calcd for C50H69N7O14 [M + Na]^+^ m/z 1014.4799, observed 1014.4721.

**Figure S38.** ^1^H NMR spectrum of **SC6** recorded in DMSO-d6.


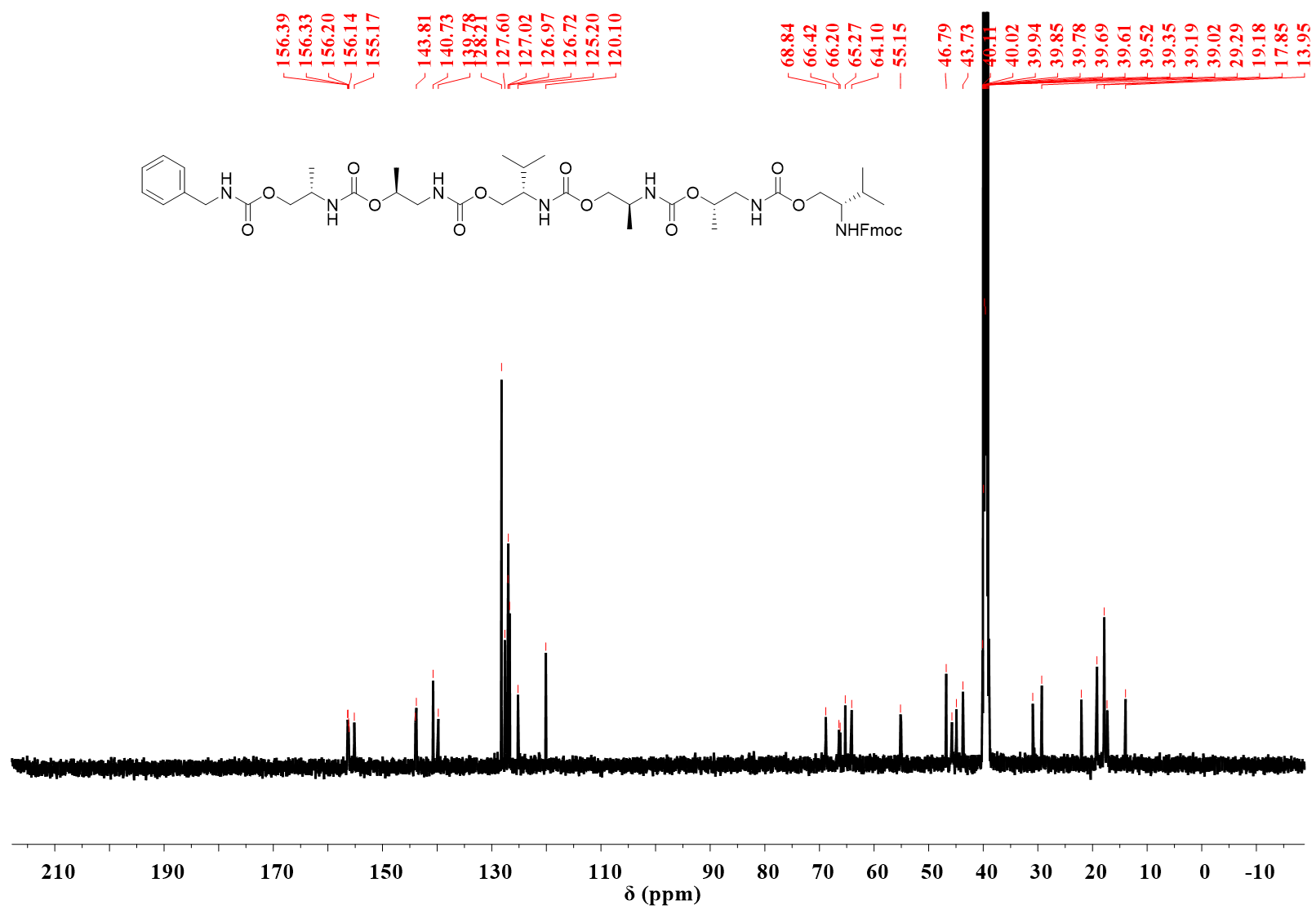


**Figure S39.** ^13^C NMR spectrum of **SC6** recorded in DMSO-d6.

**(9H-fluoren-9-yl)methyl (R)-(1-((benzylcarbamoyl)oxy)propan-2-yl)carbamate (RC1)**

^1^H NMR (500 MHz, CDCl_3_) δ 7.76 (d, *J* = 7.5 Hz, 2H), 7.59 (d, *J* = 7.4 Hz, 2H), 7.39 (t, *J* = 7.4 Hz, 2H), 7.35 – 7.25 (m, 7H), 5.00 (d, *J* = 42.9 Hz, 2H), 4.38 (dd, *J* = 11.3, 5.9 Hz, 4H), 4.21 (t, *J* = 6.8 Hz, 1H), 4.16 – 3.93 (m, 3H), 1.13 (d, *J* = 51.8 Hz, 3H).

^13^C NMR (126 MHz, CDCl_3_) δ 155.98, 155.43, 143.95, 141.34, 138.23, 128.73, 127.69, 127.61, 127.05, 125.07, 119.98, 67.59, 66.63, 47.29, 46.69, 45.19, 17.59.

**Figure S40.** ^1^H NMR spectrum of **RC1** recorded in CDCl_3_.

**Figure S41.** ^13^C NMR spectrum of **RC1** recorded in CDCl_3_.

**(R)-1-((((9H-fluoren-9-yl)methoxy)carbonyl)amino)propan-2-yl ((R)-1 ((benzylcarbamoyl)oxy)propan-2-yl)carbamate (RC2)**

^1^H NMR (500 MHz, CDCl_3_) δ 7.76 (d, *J* = 7.5 Hz, 2H), 7.59 (d, *J* = 7.4 Hz, 2H), 7.39 (t, *J* = 7.4 Hz, 2H), 7.35 – 7.26 (m, 7H), 5.21 – 4.71 (m, 3H), 4.36 (t, *J* = 13.2 Hz, 4H), 4.22 (dd, *J* = 11.9, 5.8 Hz, 1H), 4.16 – 4.02 (m, 2H), 3.95 (s, 1H), 3.56 – 3.11 (m, 2H), 1.24 (d, *J* = 13.5 Hz, 3H), 1.17 (d, *J* = 6.4 Hz, 3H).

**Figure S42.** ^1^H NMR spectrum of **RC2** recorded in CDCl_3_.

**(9H-fluoren-9-yl)methyl ((6R,10R,16R)-6,10,17-trimethyl-3,8,13-trioxo-1-phenyl-4,9,14-trioxa-2,7,12-triazaoctadecan-16-yl)carbamate (RC3)**

^1^H NMR (500 MHz, CDCl_3_) δ 7.76 (d, *J* = 7.5 Hz, 2H), 7.60 (d, *J* = 7.1 Hz, 2H), 7.39 (t, *J* = 7.4 Hz, 2H), 7.35 – 7.26 (m, 7H), 5.05 (s, 1H), 4.86 (s, 2H), 4.39 (d, *J* = 42.8 Hz, 4H), 4.14 (d, *J* = 84.2 Hz, 5H), 3.92 (s, 1H), 3.69 (s, 1H), 3.32 (s, 1H), 3.27 (s, 1H), 1.82 (s, 1H), 1.17 (d, *J* = 32.4 Hz, 6H), 0.92 (d, *J* = 32.4 Hz, 6H).

**Figure S43.** ^1^H NMR spectrum of **RC3** recorded in CDCl_3_.

**(5R,10R)-1-(9H-fluoren-9-yl)-10-isopropyl-5-methyl-3,8-dioxo-2,7-dioxa-4,9-diazaundecan-11-yl ((6R,10R)-6,10-dimethyl-3,8-dioxo-1-phenyl-4,9-dioxa-2,7-diazaundecan-11-yl)carbamate (RC4)**

^1^H NMR (500 MHz, DMSO) δ 7.89 (d, *J* = 7.5 Hz, 2H), 7.69 (t, *J* = 8.3 Hz, 3H), 7.42 (t, *J* = 7.4 Hz, 2H), 7.36 – 7.28 (m, 4H), 7.23 (dd, *J* = 14.7, 7.1 Hz, 4H), 7.17 (t, *J* = 5.3 Hz, 1H), 7.04 (d, *J* = 9.0 Hz, 1H), 6.96 (d, *J* = 7.5 Hz, 1H), 4.65 (dd, *J* = 12.3, 6.1 Hz, 1H), 4.40 – 4.12 (m, 5H), 4.02 (dd, *J* = 10.9, 4.6 Hz, 1H), 3.96 – 3.63 (m, 7H), 3.50 (t, *J* = 13.8 Hz, 1H), 3.06 (d, *J* = 5.3 Hz, 2H), 1.72 (dd, *J* = 12.9, 6.5 Hz, 1H), 1.19 – 0.92 (m, 9H), 0.82 (d, *J* = 18.5 Hz, 6H).

**Figure S44.** ^1^H NMR spectrum of **RC4** recorded in DMSO-d6.

**(6R,10R,15R)-1-(9H-fluoren-9-yl)-15-isopropyl-6,10-dimethyl-3,8,13-trioxo-2,7,12-trioxa-4,9,14-triazahexadecan-16-yl ((6R,10R)-6,10-dimethyl-3,8-dioxo-1-phenyl-4,9-dioxa-2,7-diazaundecan-11-yl)carbamate (RC5)**

^1^H NMR (500 MHz, DMSO) δ 7.91 (d, *J* = 7.5 Hz, 2H), 7.70 (d, *J* = 6.4 Hz, 3H), 7.43 (t, *J* = 7.3 Hz, 3H), 7.33 (dd, *J* = 17.1, 7.4 Hz, 4H), 7.28 – 7.22 (m, 3H), 7.20 (s, 1H), 7.05 (d, *J* = 8.9 Hz, 1H), 6.97 (d, *J* = 5.9 Hz, 2H), 4.68 (dt, *J* = 19.4, 6.0 Hz, 2H), 4.31 (d, *J* = 5.8 Hz, 2H), 4.26 – 4.22 (m, 1H), 4.19 (d, *J* = 5.8 Hz, 2H), 4.04 (dd, *J* = 10.8, 4.1 Hz, 1H), 3.93 (dd, *J* = 9.9, 6.7 Hz, 1H), 3.90 – 3.81 (m, 3H), 3.73 (dt, *J* = 13.0, 8.0 Hz, 3H), 3.55 – 3.48 (m, 1H), 3.17 – 3.04 (m, 4H), 1.74 (td, *J* = 12.7, 6.3 Hz, 1H), 1.11 (d, *J* = 4.7 Hz, 6H), 1.07 – 1.00 (m, 6H), 0.85 (dd, *J* = 10.9, 6.6 Hz, 6H).

**Figure S45.** ^1^H NMR spectrum of **RC5** recorded in DMSO-d6.

**(5R,11R,15R,20R)-1-(9H-fluoren-9-yl)-5,20-diisopropyl-11,15-dimethyl-3,8,13,18-tetraoxo-2,7,12,17-tetraoxa-4,9,14,19-tetraazahenicosan-21-yl ((6R,10R)-6,10-dimethyl-3,8-dioxo-1-phenyl-4,9-dioxa-2,7-diazaundecan-11-yl)carbamate (RC6)**

^1^H NMR (500 MHz, DMSO) δ 7.91 (d, *J* = 7.5 Hz, 2H), 7.75 – 7.67 (m, 3H), 7.43 (t, *J* = 7.4 Hz, 2H), 7.37 – 7.30 (m, 4H), 7.25 (dd, *J* = 14.8, 7.4 Hz, 4H), 7.19 (d, *J* = 5.5 Hz, 2H), 7.05 (d, *J* = 8.8 Hz, 1H), 6.96 (dd, *J* = 14.0, 7.9 Hz, 2H), 4.67 (dt, *J* = 11.8, 6.0 Hz, 2H), 4.37 (dd, *J* = 12.8, 9.9 Hz, 1H), 4.23 (t, *J* = 6.4 Hz, 2H), 4.19 (d, *J* = 6.1 Hz, 2H), 4.05 (td, *J* = 11.4, 4.7 Hz, 2H), 3.96 – 3.81 (m, 5H), 3.73 (tt, *J* = 12.9, 6.3 Hz, 3H), 3.60 – 3.46 (m, 2H), 3.15 – 3.01 (m, 4H), 1.85 – 1.65 (m, 2H), 1.11 (d, *J* = 5.8 Hz, 6H), 1.04 (t, *J* = 6.0 Hz, 6H), 0.86 (dd, *J* = 17.6, 7.5 Hz, 12H).

^13^C NMR (126 MHz, DMSO) δ: 156.79, 128.70, 128.09, 127.48, 125.69, 120.59, 69.31, 66.91, 64.59, 39.85, 39.69, 39.52, 29.79, 19.67, 18.34, 17.83.

HRMS calcd for C50H69N7O14 [M + Na]+ m/z 1014.4799, observed 1014.4824.

**Figure S46.** ^1^H NMR spectrum of **RC6** recorded in DMSO-d6.

**Figure S47.** ^13^C NMR spectrum of **RC6** recorded in DMSO-d6.

**(9H-fluoren-9-yl)methyl (S)-(1-((benzylcarbamoyl)oxy)propan-2-yl)carbamate (SRC1)**

^1^H NMR (500 MHz, DMSO) δ 7.89 (d, *J* = 7.5 Hz, 2H), 7.69 (t, *J* = 5.9 Hz, 3H), 7.41 (t, *J* = 7.4 Hz, 2H), 7.27 (dtd, *J* = 22.8, 15.2, 7.3 Hz, 8H), 4.36 – 4.11 (m, 5H), 3.88 (d, *J* = 6.1 Hz, 2H), 3.73 (dt, *J* = 13.5, 6.7 Hz, 1H), 1.06-1.04 (d, 3H).

**Figure S48.** ^1^H NMR spectrum of **SRC1** recorded in DMSO-d6.

**(5R,10S)-1-(9H-fluoren-9-yl)-5,10-dimethyl-3,8-dioxo-2,7-dioxa-4,9-diazaundecan-11-yl benzylcarbamate (SRC2)**

^1^H NMR (500 MHz, DMSO) δ 7.91 (d, *J* = 7.5 Hz, 2H), 7.71 (d, *J* = 6.1 Hz, 3H), 7.43 (t, *J* = 7.4 Hz, 2H), 7.39 – 7.30 (m, 4H), 7.24 (dd, *J* = 15.3, 7.3 Hz, 4H), 7.14 (d, *J* = 7.7 Hz, 1H), 4.32 (p, *J* = 10.3 Hz, 2H), 4.23 (t, *J* = 6.7 Hz, 1H), 4.18 (d, *J* = 6.2 Hz, 2H), 3.87 (d, *J* = 6.3 Hz, 4H), 3.79 – 3.67 (m, 2H), 1.06-1.04 (d, 6H).

**Figure S49.** ^1^H NMR spectrum of **SRC2** recorded in DMSO-d6.

**(6S,10R,15S)-1-(9H-fluoren-9-yl)-6,10,15-trimethyl-3,8,13-trioxo-2,7,12-trioxa-4,9,14-triazahexadecan-16-yl benzylcarbamate (SRC3)**

1H NMR (500 MHz, DMSO) δ 7.91 (d, *J* = 7.5 Hz, 2H), 7.71 (d, *J* = 7.2 Hz, 3H), 7.43 (t, *J* = 7.4 Hz, 3H), 7.37 – 7.29 (m, 4H), 7.24 (dd, *J* = 14.3, 7.1 Hz, 3H), 7.14 (s, 1H), 7.03 (s, 1H), 4.68 (d, *J* = 6.1 Hz, 1H), 4.32 (d, *J* = 6.6 Hz, 2H), 4.23 (t, *J* = 6.9 Hz, 1H), 4.18 (d, *J* = 6.1 Hz, 2H), 3.85 (dd, *J* = 19.8, 5.4 Hz, 4H), 3.74 (td, *J* = 11.2, 4.7 Hz, 2H), 3.20 – 3.01 (m, 2H), 1.12-1.11 (s, 3H), 1.06-1.04 (s, 6H).

**Figure S50.** ^1^H NMR spectrum of **SRC3** recorded in DMSO-d6.

**(6R,11S,15R,20S)-1-(9H-fluoren-9-yl)-6,11,15,20-tetramethyl-3,8,13,18-tetraoxo-2,7,12,17-tetraoxa-4,9,14,19-tetraazahenicosan-21-yl benzylcarbamate (SRC4)**

^1^H NMR (500 MHz, DMSO) δ 7.91 (d, *J* = 7.5 Hz, 2H), 7.70 (d, *J* = 7.2 Hz, 3H), 7.43 (dd, *J* = 13.8, 6.3 Hz, 3H), 7.36 – 7.30 (m, 4H), 7.24 (dd, *J* = 14.3, 7.1 Hz, 3H), 7.13 (s, 2H), 7.00 (s, 1H), 4.75 – 4.63 (m, 2H), 4.31 (d, *J* = 7.0 Hz, 2H), 4.23 (t, *J* = 7.0 Hz, 1H), 4.19 (d, *J* = 6.1 Hz, 2H), 3.87 (d, *J* = 6.3 Hz, 2H), 3.83 (s, 2H), 3.78 – 3.68 (m, 2H), 3.17 – 3.03 (m, 4H), 1.11 (d, *J* = 6.3 Hz, 6H), 1.05 (d, *J* = 6.5 Hz, 6H).

**Figure S51.** ^1^H NMR spectrum of **SRC4** recorded in DMSO-d6.

**(9H-fluoren-9-yl)methyl ((6S,11R,15S,20R,26S)-6,11,15,20,27-pentamethyl-3,8,13,18,23-pentaoxo-1-phenyl-4,9,14,19,24-pentaoxa-2,7,12,17,22-pentaazaoctacosan-26-yl)carbamate (SRC5)**

^1^H NMR (500 MHz, DMSO) δ 7.91 (d, *J* = 7.5 Hz, 2H), 7.69 (d, *J* = 6.1 Hz, 3H), 7.43 (t, *J* = 7.4 Hz, 2H), 7.38 – 7.30 (m, 4H), 7.28 – 7.21 (m, 4H), 7.19 (s, 1H), 7.11 (d, *J* = 19.8 Hz, 2H), 7.00 (s, 1H), 4.74 – 4.60 (m, 2H), 4.36 (dd, *J* = 12.8, 9.5 Hz, 1H), 4.24 (q, *J* = 7.1 Hz, 2H), 4.19 (d, *J* = 6.1 Hz, 2H), 4.05 (dd, *J* = 11.1, 4.6 Hz, 1H), 3.87 (dd, *J* = 20.2, 13.8 Hz, 5H), 3.72 (dd, *J* = 13.7, 7.1 Hz, 2H), 3.54 (s, 1H), 3.08 (d, *J* = 5.2 Hz, 4H), 1.77 (dd, *J* = 12.8, 6.4 Hz, 1H), 1.11 (d, *J* = 6.1 Hz, 6H), 1.05 (d, *J* = 6.4 Hz, 6H), 0.86 (dd, *J* = 9.6, 7.0 Hz, 6H).

**Figure S52.** ^1^H NMR spectrum of **SRC5** recorded in DMSO-d6.

**(5R,10S)-1-(9H-fluoren-9-yl)-5,10-diisopropyl-3,8-dioxo-2,7-dioxa-4,9-diazaundecan-11-yl ((6S,11R,15S,20R)-6,11,15,20-tetramethyl-3,8,13,18-tetraoxo-1-phenyl-4,9,14,19-tetraoxa-2,7,12,17-tetraazahenicosan-21-yl)carbamate (SRC6)**

^1^H NMR (500 MHz, DMSO) δ 7.91 (d, *J* = 7.5 Hz, 2H), 7.75 – 7.61 (m, 3H), 7.43 (t, *J* = 7.4 Hz, 2H), 7.34 (ddd, *J* = 13.1, 7.4, 2.7 Hz, 4H), 7.28 – 7.21 (m, 4H), 7.16 – 7.05 (m, 2H), 7.01 (d, *J* = 8.7 Hz, 2H), 6.76 (dd, *J* = 54.9, 33.6 Hz, 1H), 4.67 (dd, *J* = 11.8, 5.8 Hz, 2H), 4.39 (t, *J* = 11.3 Hz, 1H), 4.22 (dd, *J* = 28.1, 6.4 Hz, 4H), 4.10 (dd, *J* = 11.0, 4.8 Hz, 1H), 4.01 (dd, *J* = 10.9, 4.7 Hz, 1H), 3.88 (d, *J* = 6.3 Hz, 2H), 3.85 (s, 3H), 3.80 – 3.68 (m, 3H), 3.56 (dd, *J* = 23.0, 14.5 Hz, 2H), 3.09 (s, 4H), 1.76 (dt, *J* = 22.2, 6.6 Hz, 2H), 1.14 – 1.09 (m, 6H), 1.08 – 1.03 (m, 6H), 0.86 (ddd, *J* = 16.3, 10.2, 5.1 Hz, 12H).

^13^C NMR (126 MHz, DMSO) δ 156.85, 156.62, 156.44, 156.01, 155.58, 144.30, 141.23, 128.70, 128.09, 127.49, 127.21, 125.67, 120.58, 69.48, 66.87, 65.75, 64.55, 55.55, 46.33, 45.29, 44.23, 39.85, 39.69, 39.52, 29.78, 19.67, 18.34, 17.80.

HRMS calcd for C50H69N7O14 [M + Na]+ m/z 1014.4799, observed 1014.4819.

**Figure S53.** ^1^H NMR spectrum of **SRC6** recorded in DMSO-d6.

**Figure S54.** ^13^C NMR spectrum of **SRC6** recorded in DMSO-d6.

**Figure S55.** ESI-MS spectrum of **SC6** (Calculated [M+Na]^+^: 1014.4799, observed [M+Na]^+^: 1014.4821).

**Figure S56.** ESI-MS spectrum of **RC6** (Calculated [M+Na]^+^: 1014.4799, observed [M+Na]^+^: 1014.4824).

**Figure S57.** ESI-MS spectrum of **SRC6** (Calculated [M+Na]^+^: 1014.4799, observed [M+Na]^+^: 1014.4819).


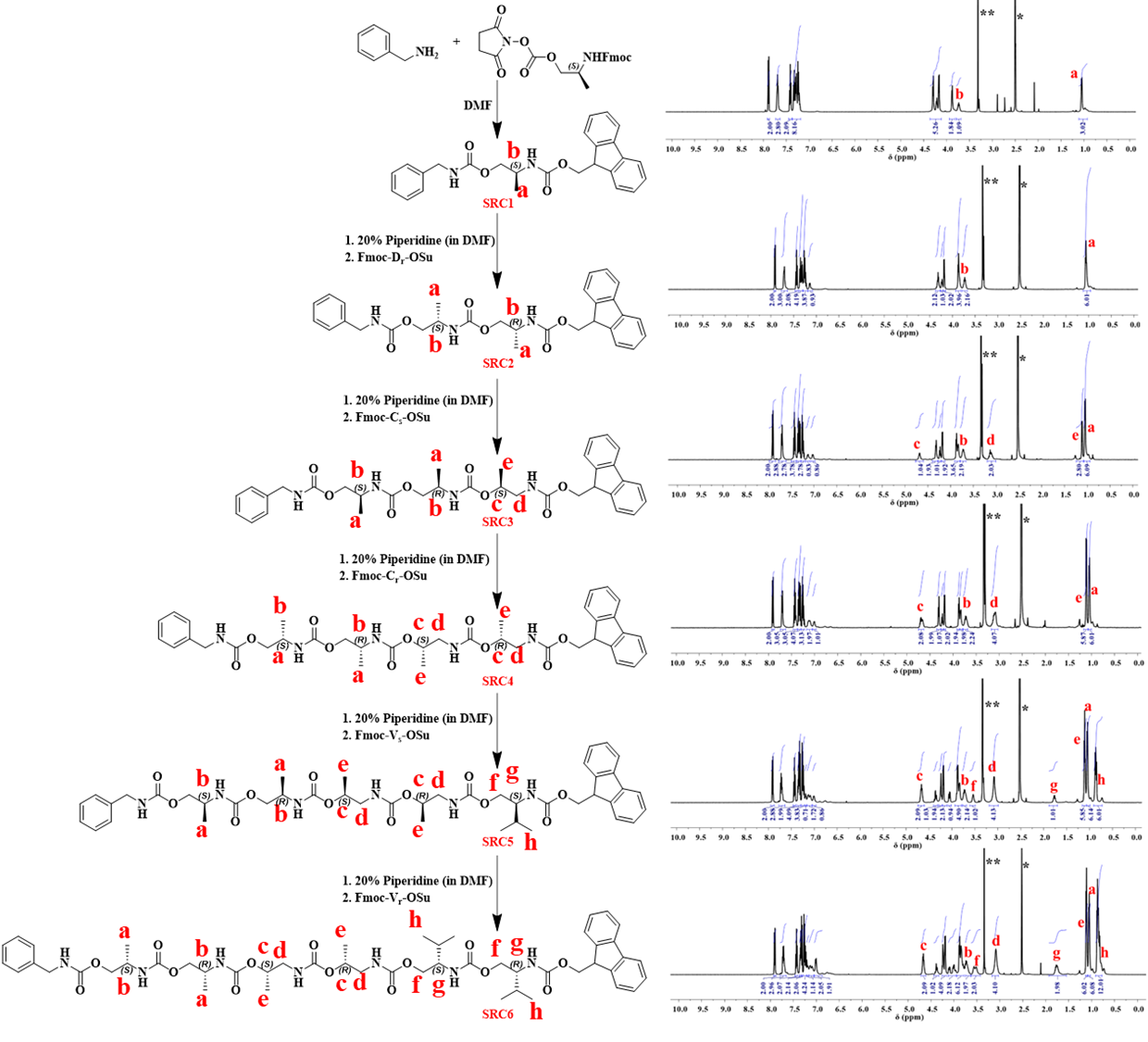


**Figure S58.** Synthetic scheme for **SRC6** along with ^1^H NMR analysis of each sequence in DMSO-d6.

**Figure S59.** TGA spectra of **SC6**, **RC6**, and **SRC6** sequences

#### **Table S4.** Thermal Properties of **SC6**, **RC6**, and **SRC6**

| Sample | Td95 (°C) | Td90 (°C) | Td20 (°C) | Tg (1st Heat) (°C) | Tg (2nd Heat) (°C) | Tc (1st Heat) (°C) | Tc (2nd Heat) (°C) | Tm (1st Heat) (°C) | Tm (2nd Heat) (°C) | ΔH (1st Heat) (J/g) | ΔH (1st Heat) (J/g) |
| --- | --- | --- | --- | --- | --- | --- | --- | --- | --- | --- | --- |
| SC6 | 206 | 216 | 277 | 77 | 53.77 | 98 | - | 141 | - | 29.08 | - |
| RC6 | 191 | 213 | 273 | 71 | 33.87 | 97 | 96 | 144 | 115.92 | 34.5 | 0.32 |
| SRC6 | 193 | 212 | 266 | 71.9 | 34.61 | 97 | 94.6, 117.78 | 137.5 | 139.06 | 33.5 | 9.39 |


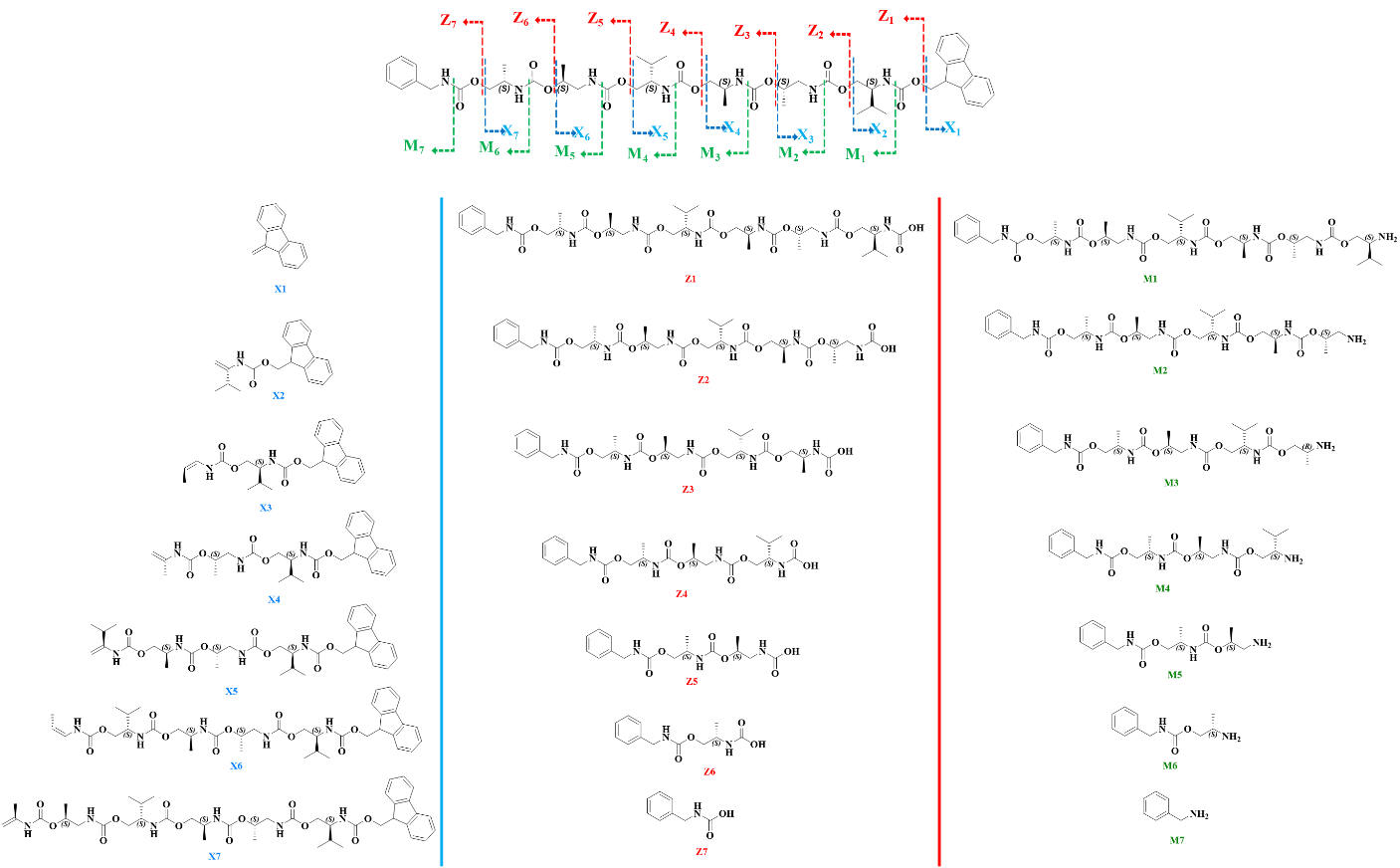


**Figure S60.** Detailed fragmentation pattern of **SC6** in Tandem MS (MS/MS) study.


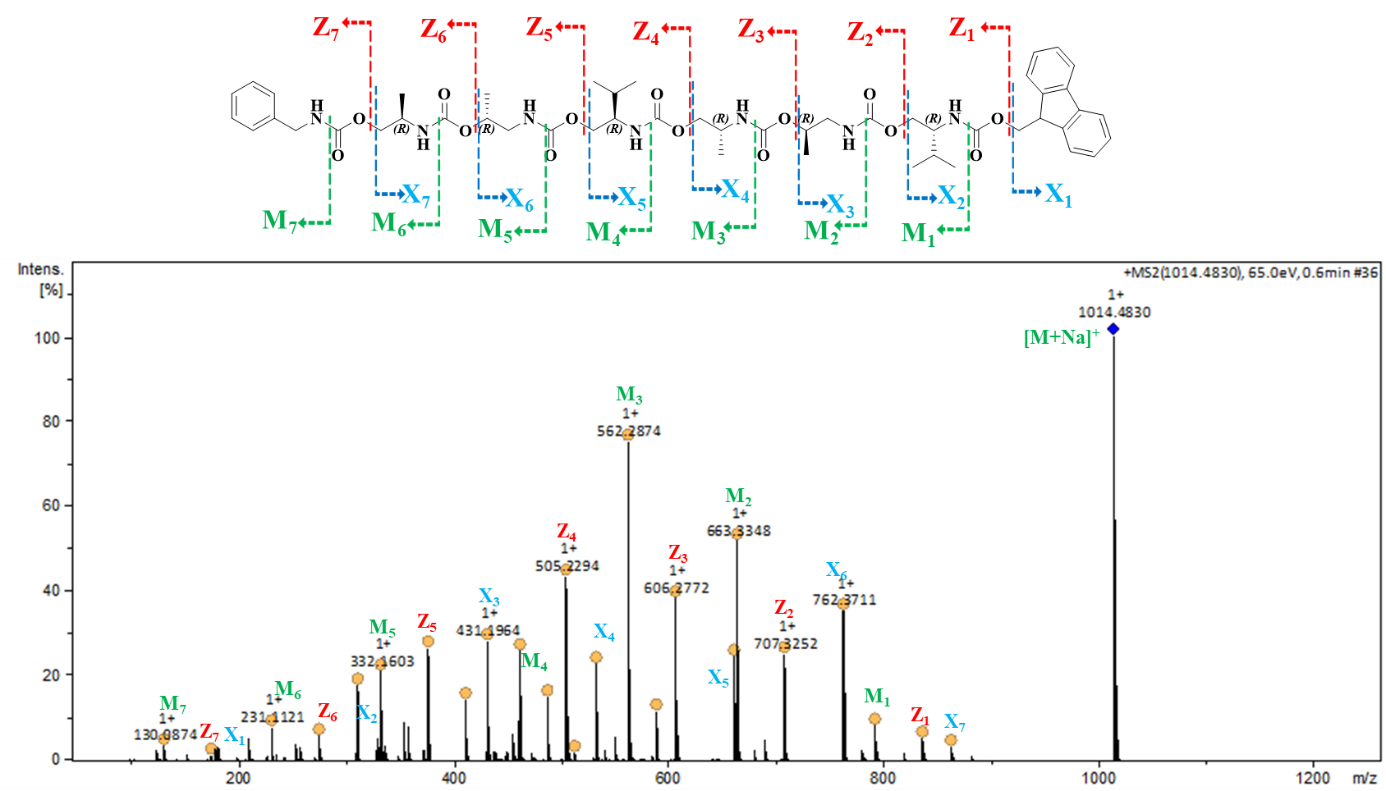


**Figure S61.** Tandem MS(MS/MS) spectrum of **RC6**.


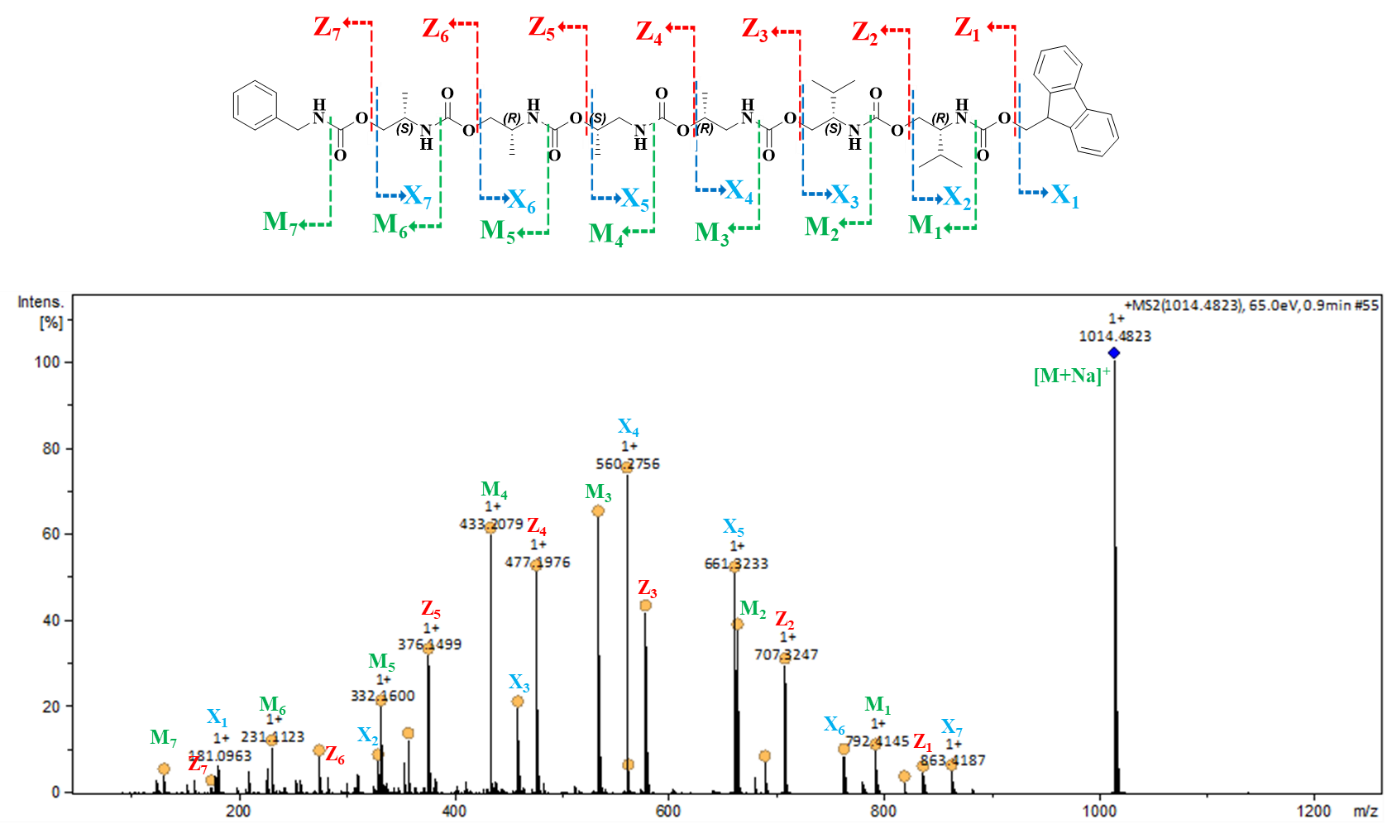


**Figure S62.** Tandem MS(MS/MS) spectrum of **SRC6**.

**Figure S63.** (A) SEC analysis of S stereospecific SDOU and (B) ESI-MS analysis of **SC7** (Calculated [M+Na]^+^: 1025:4732, observed [M+Na]^+^: 1025.4617).


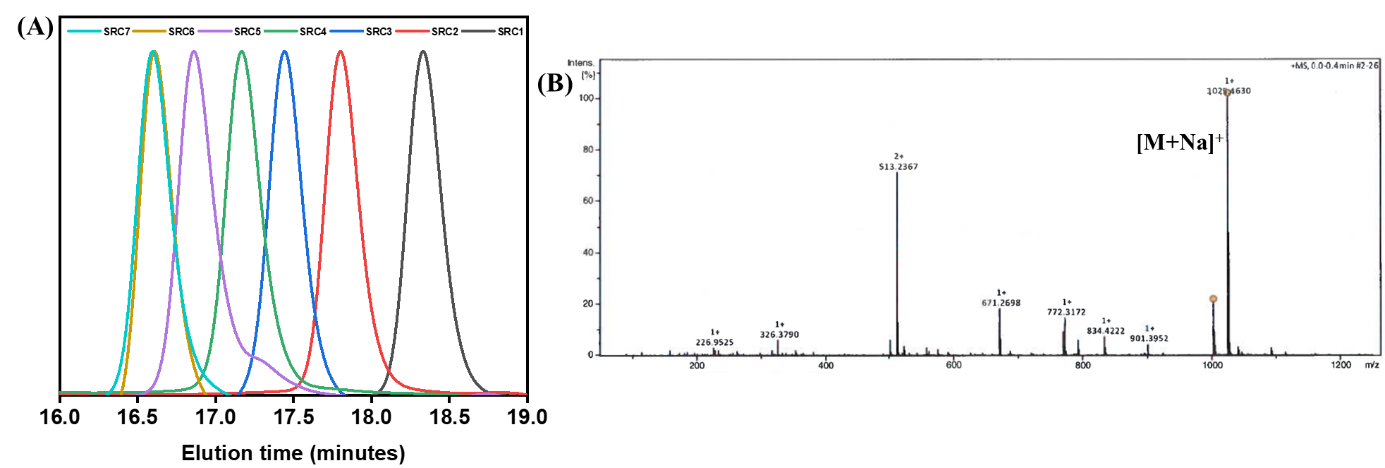


**Figure S64.** SEC analysis of SR stereospecific SDOU and (B) ESI-MS analysis of **SRC7** (Calculated [M+Na]^+^: 1025:4732, observed [M+Na]^+^: 1025.4630).


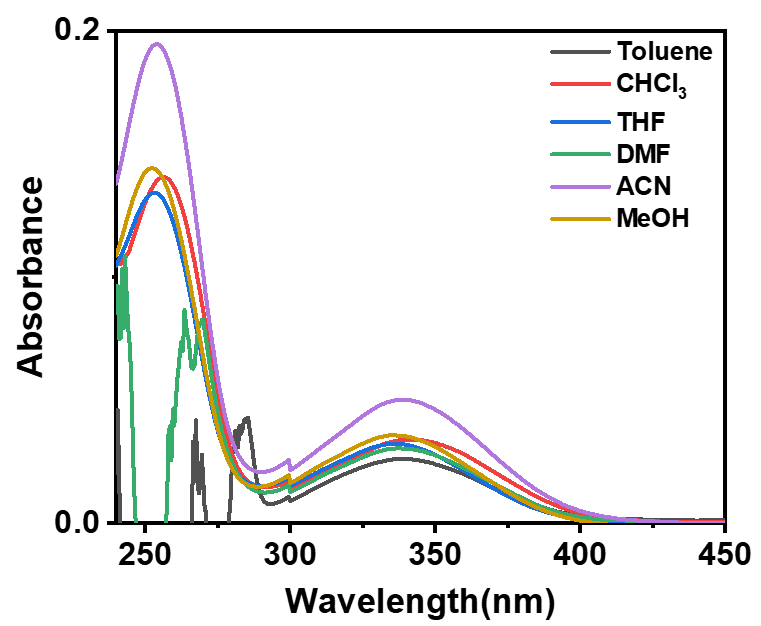


**Figure S65.** UV-Vis spectra of **SC7** (10 µM) in different solvent.


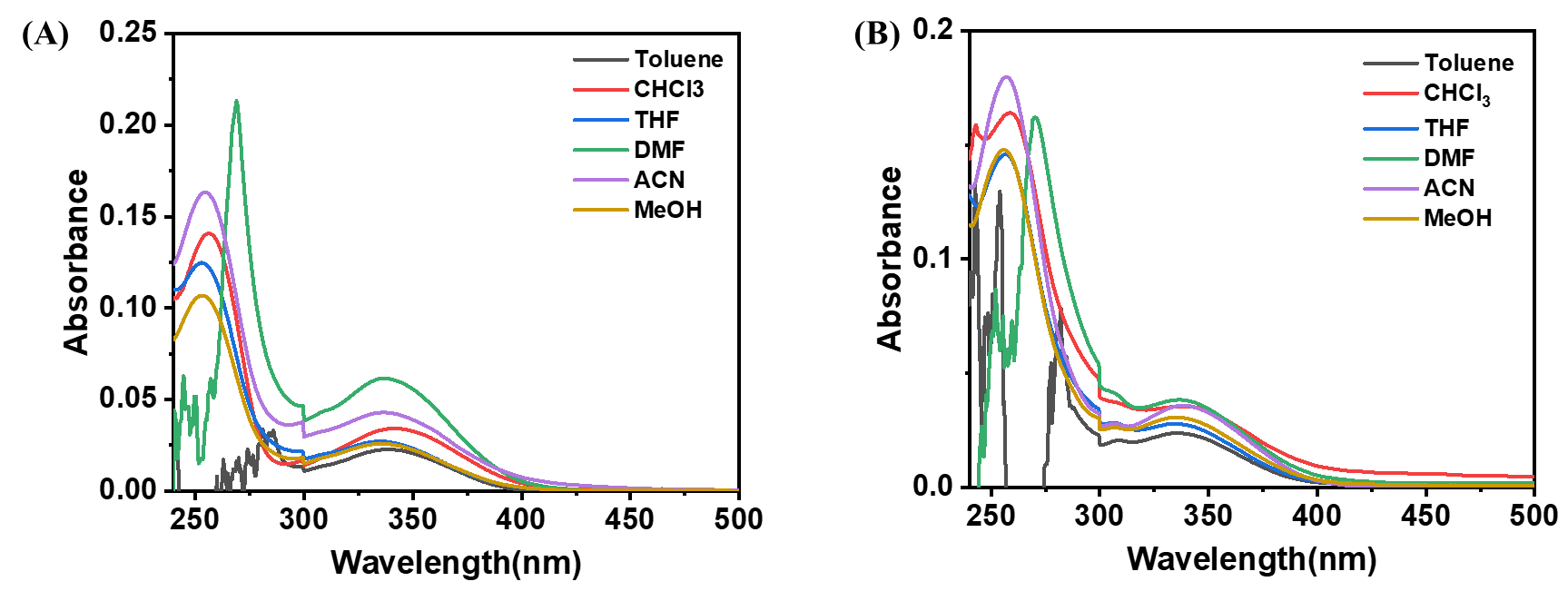


**Figure S66.** UV-Vis spectra of (A**) RC7** (10 µM) and (B) **SRC7** (10 µM) in different solvents.

**Table S5**: Observed stokes shift for **SC7** in different solvent mediums.

| **Solvent** | **λ_abs_ (nm)** | **λ_ems_ (nm)** | **Stokes shift (nm)** |
| --- | --- | --- | --- |
| **Toluene** | **338** | **473** | **135** |
| **CHCl_3_** | **343** | **489** | **146** |
| **THF** | **328** | **489** | **161** |
| **DMF** | **336** | **496** | **160** |
| **ACN** | **338** | **512** | **174** |
| **MeOH** | **336** | **513** | **177** |


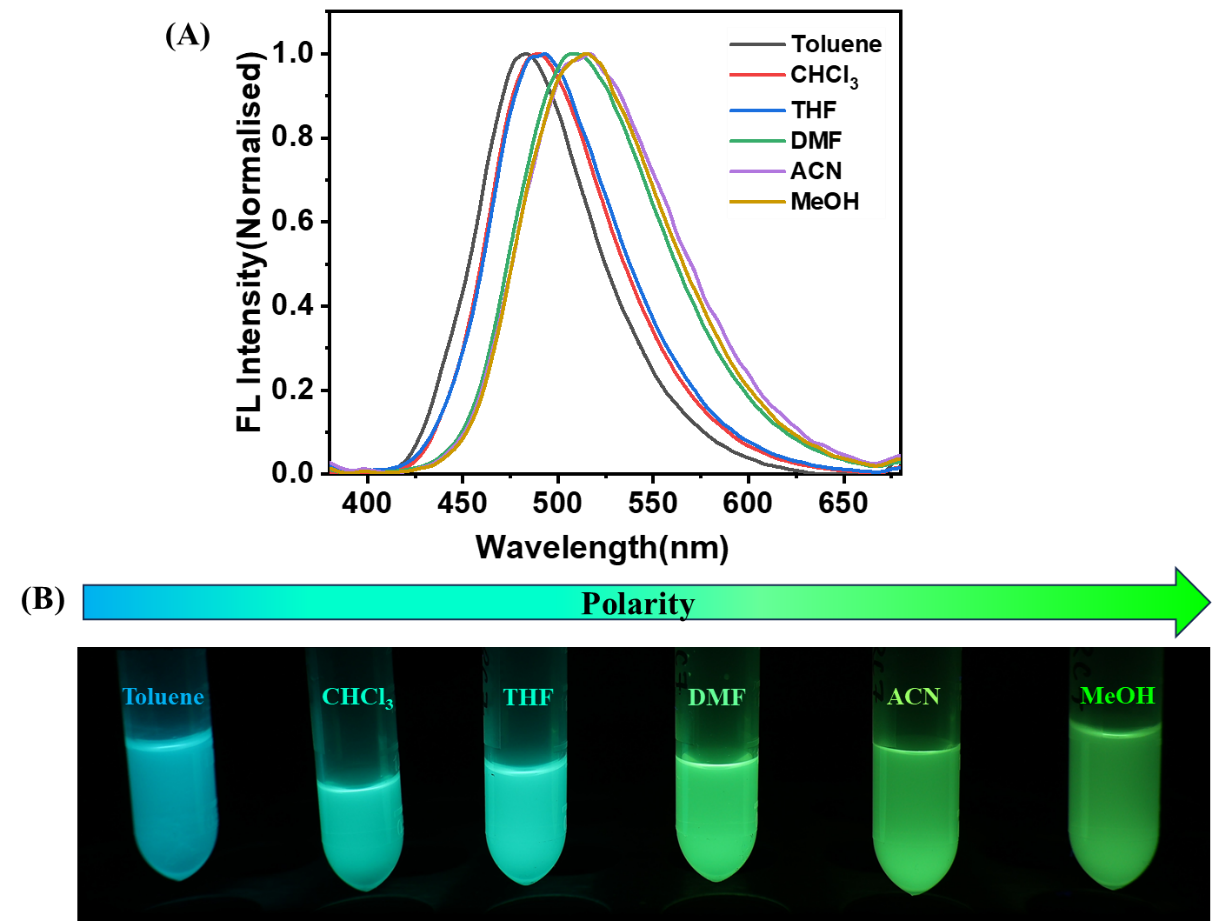


**Figure S67.** (A) Emission spectra of **RC7** (10 µM) in different solvents and (B) Pictorial representation of **RC7** solutions in different solvents under hand held UV light. (λ_ex_ =340 nm)


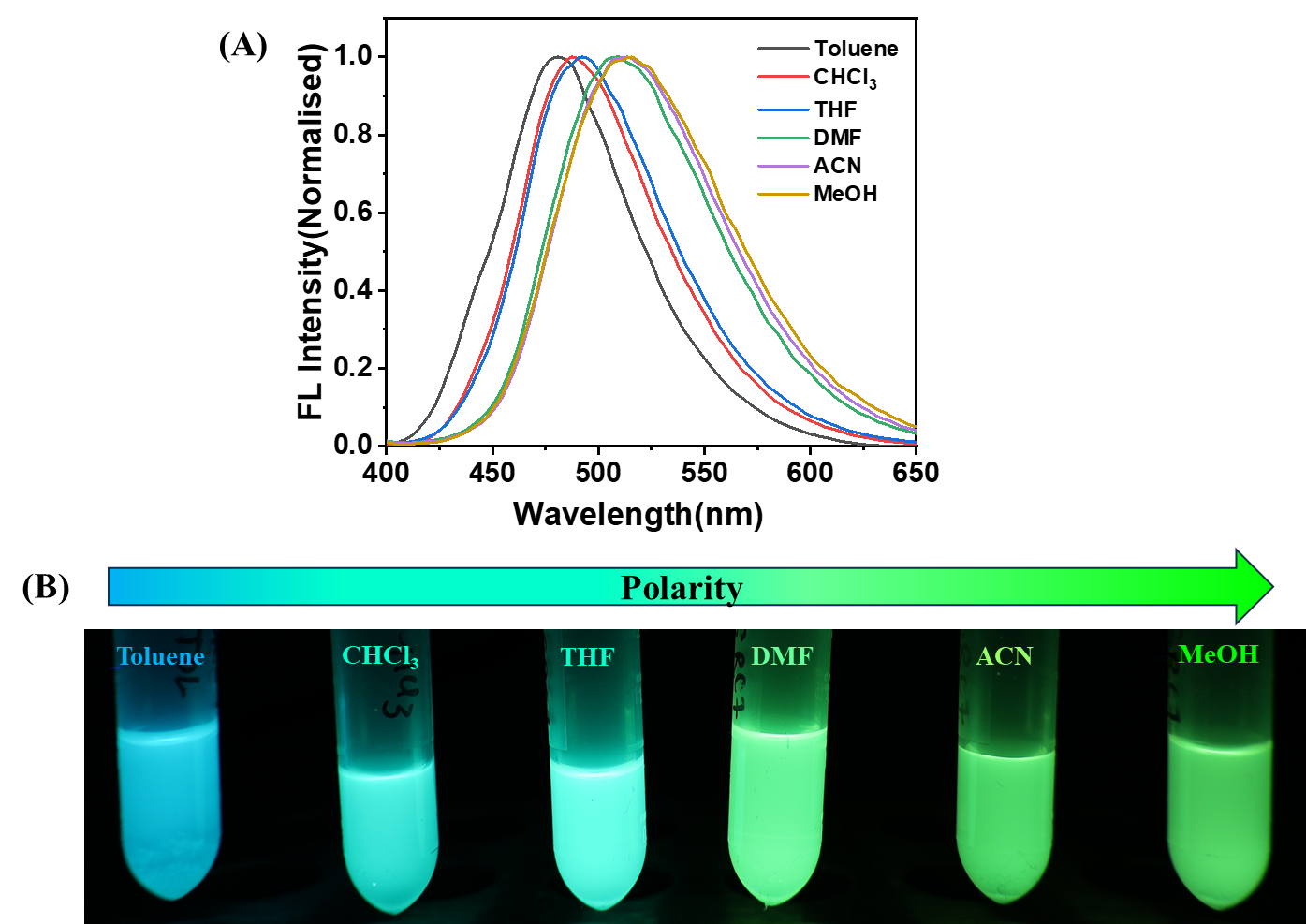


**Figure S68.** (A) Emission spectra of **SRC7** (10 µM) in different solvents and (B) Pictorial representation of **SRC7** solutions in different solvents under hand held UV light. (λ_ex_ =340 nm)


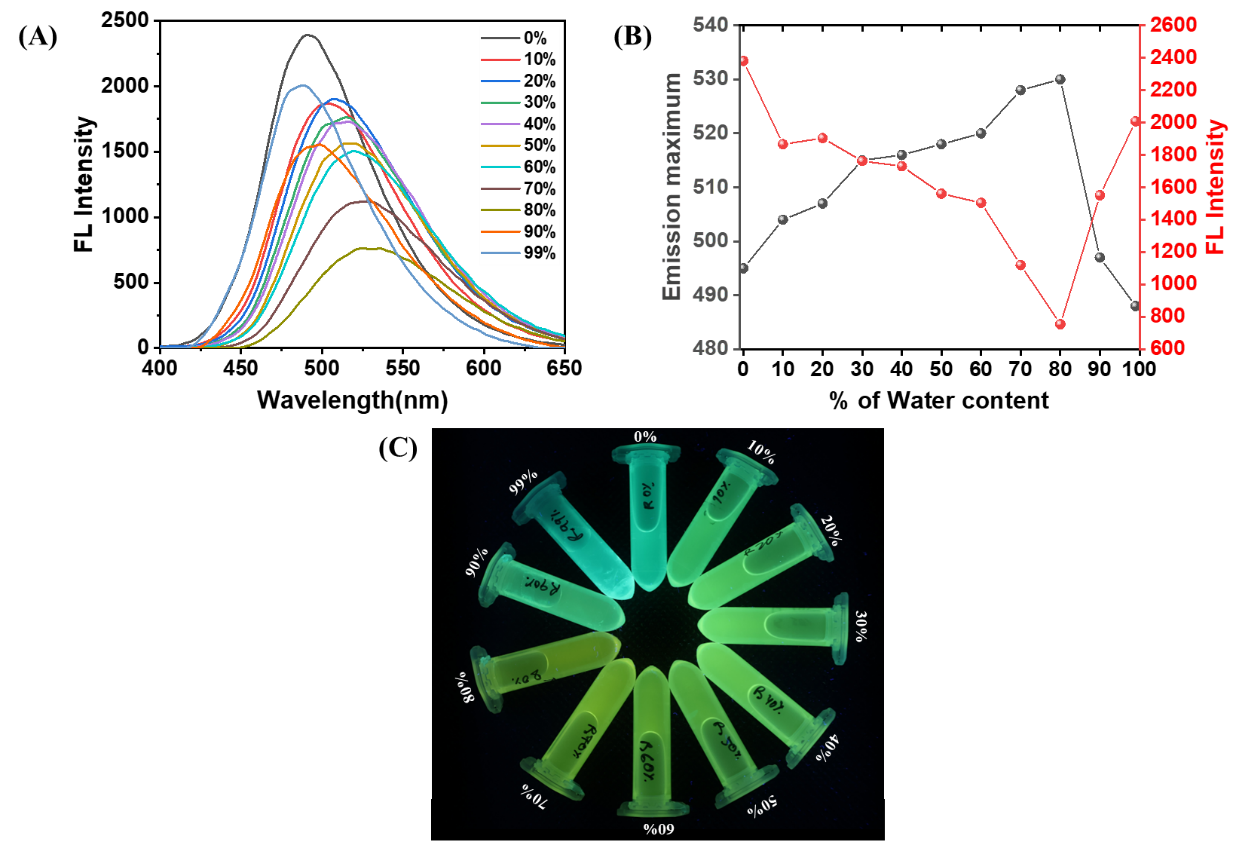


**Figure S69.** **(A)** Fluorescence spectroscopic study of **RC7** (10 µM) with changing water content in THF; **(B)** Plot of emission maxima and emission intensity with changing water content for **RC7**; **(C)** Pictorial representation of **RC7** solutions for different THF-water content under hand held UV light.


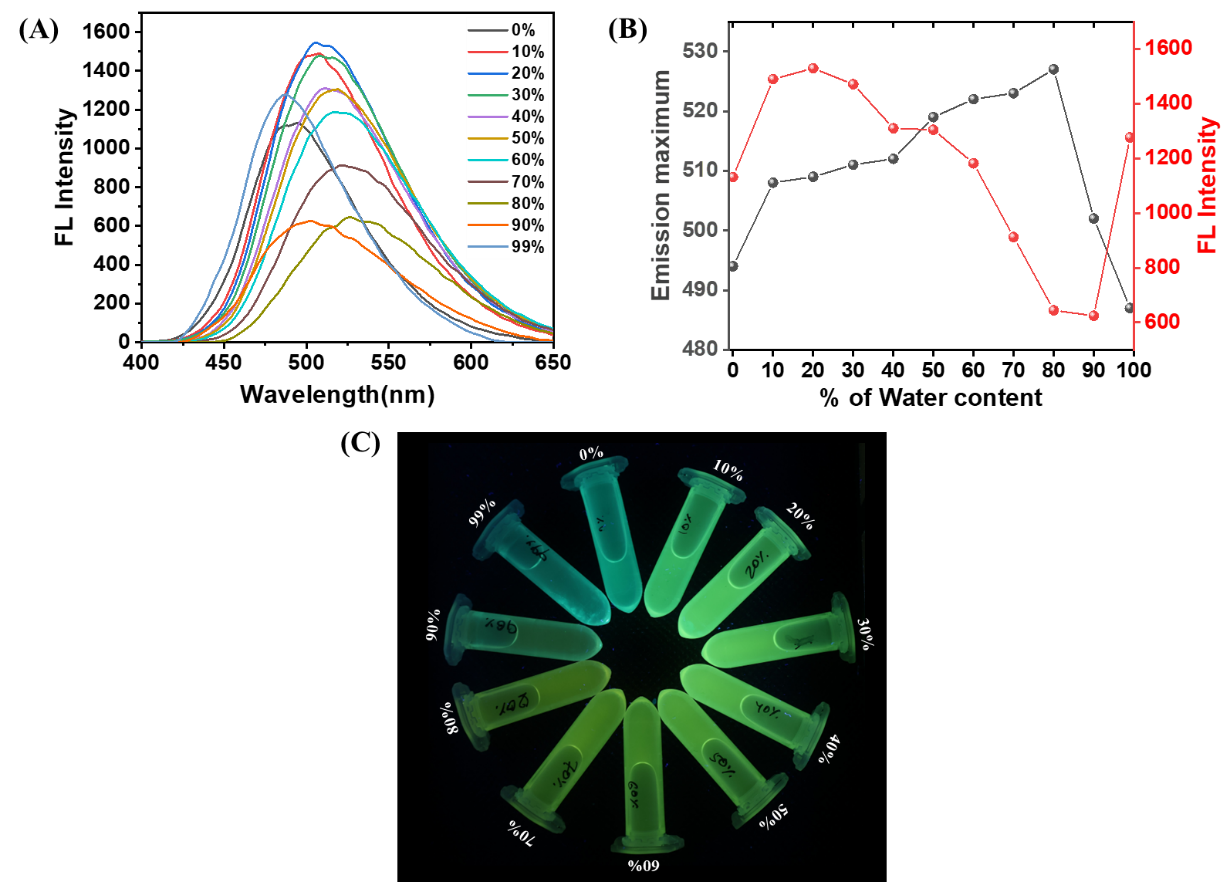


**Figure S70.** **(A)** Fluorescence spectroscopic study of **SRC7** (10 µM) with changing water content in THF; **(B)** Plot of emission maxima and emission intensity with changing water content for **SRC7**; **(C)** Pictorial representation of **SRC7** solutions for different THF-water content under hand held UV light.


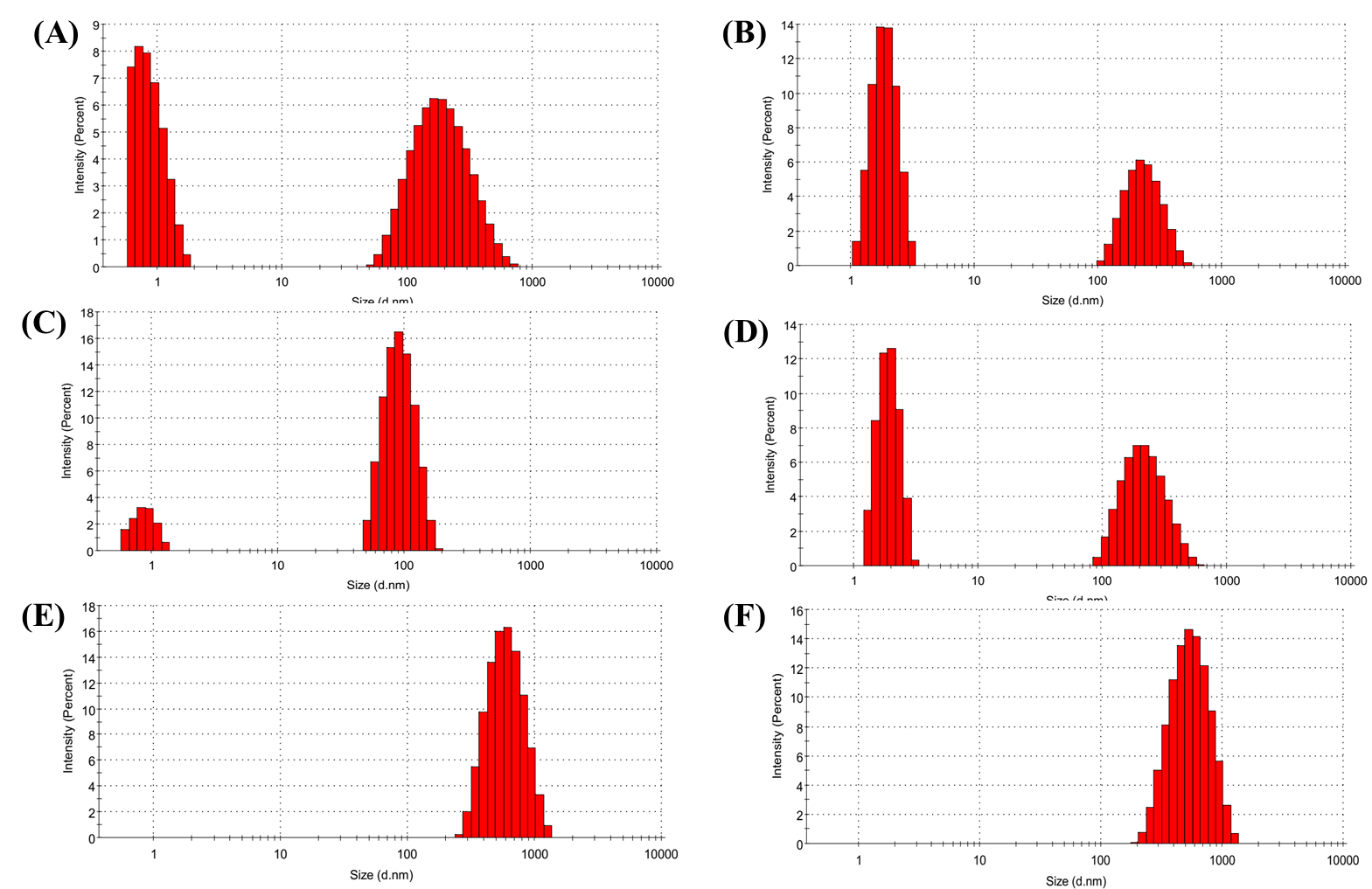


**Figure S71.** Particle size distribution using DLS for **SC7** in THF-water mixture: **(A)** 10% Water; **(B)** 30% Water; **(C)** 50% Water; **(D)** 80% Water; **(E)** 90% Water and **(F)** 99% Water.


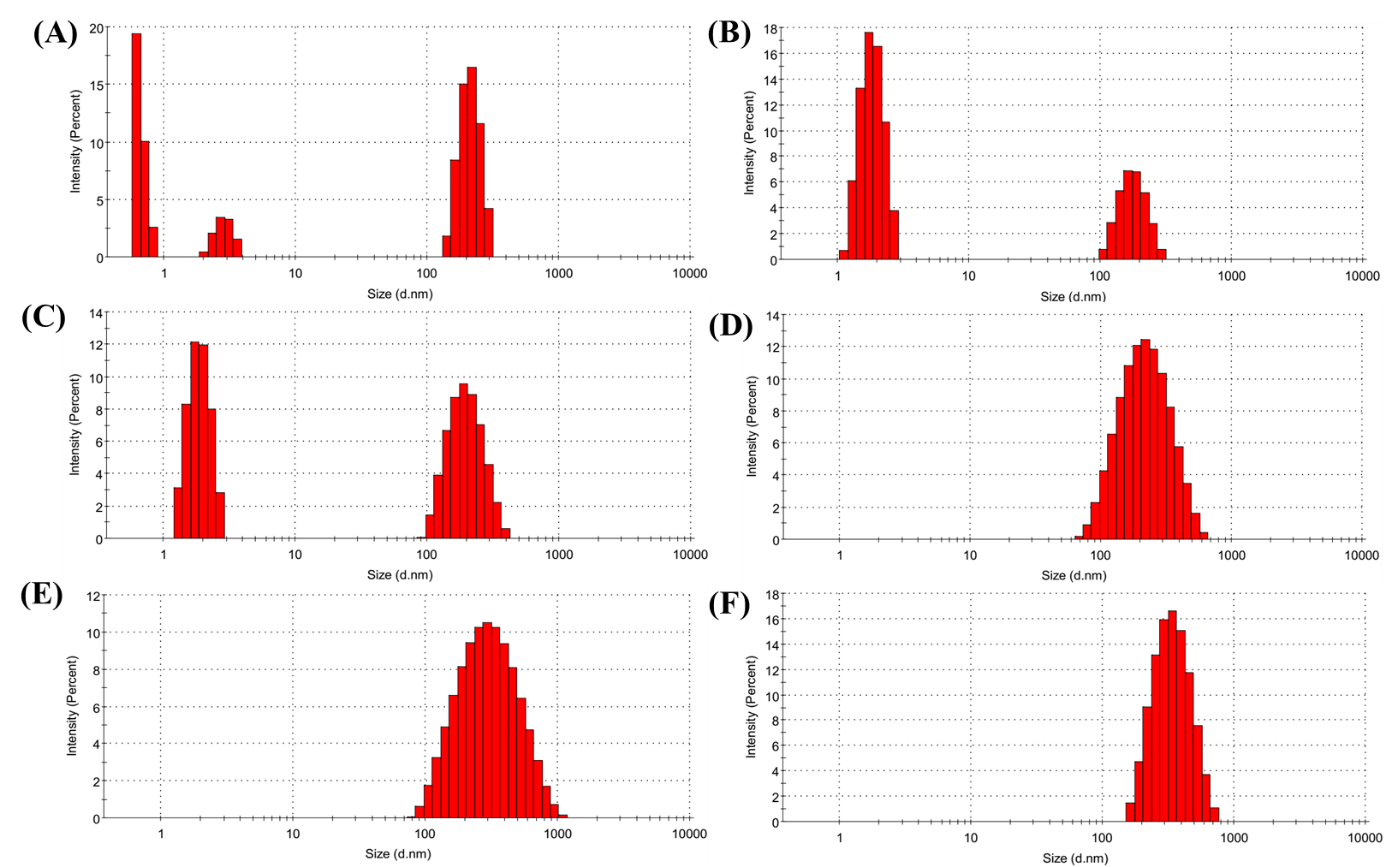


**Figure S72.** Particle size distribution using DLS for **RC7** in THF-water mixture: **(A)** 10% Water; **(B)** 30% Water; **(C)** 50% Water; **(D)** 80% Water; **(E)** 90% Water and **(F)** 99% Water.


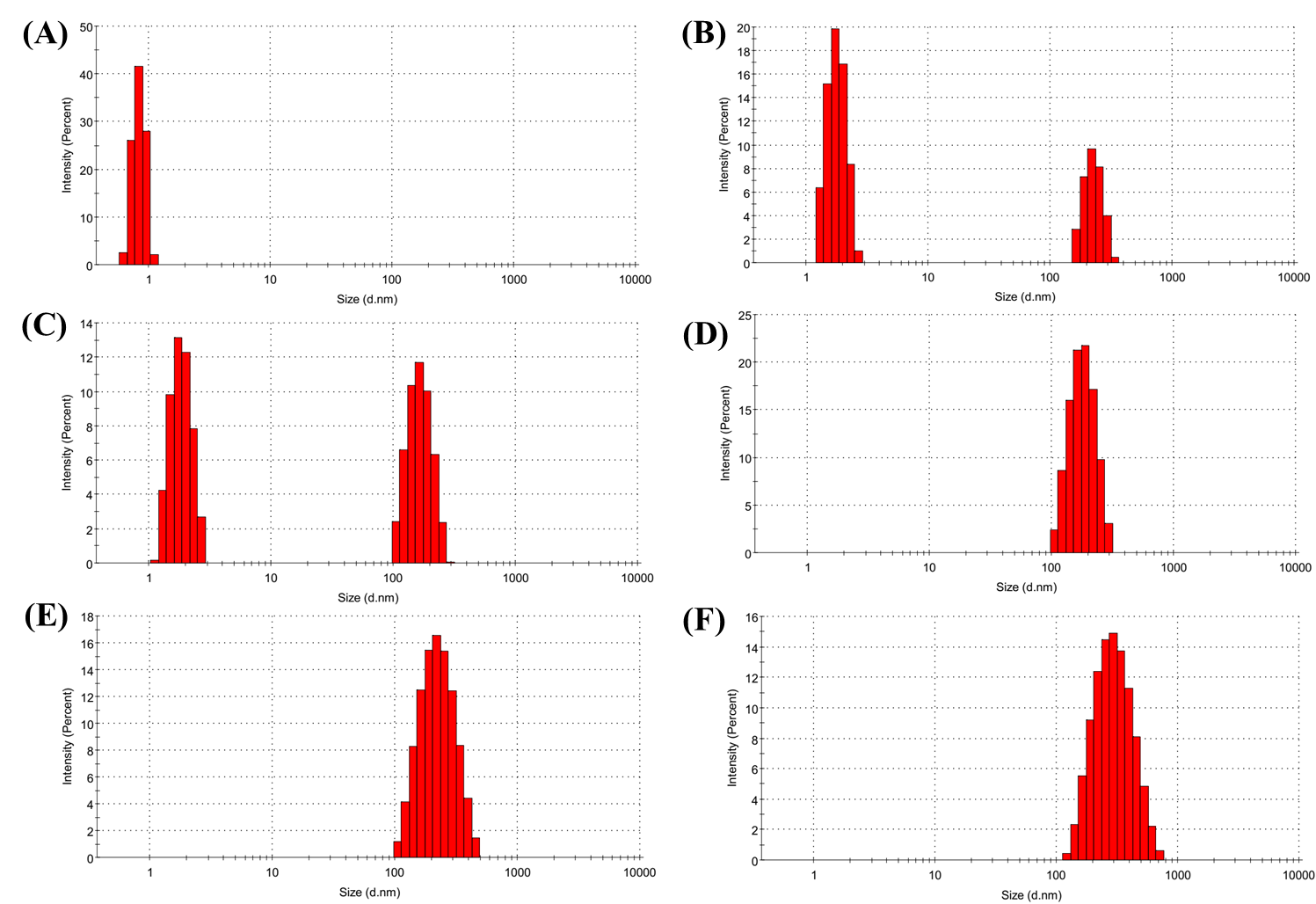


**Figure S73.** Particle size distribution using DLS for **SRC7** in THF-water mixture: **(A)** 10% Water; **(B)** 30% Water; **(C)** 50% Water; **(D)** 80% Water; **(E)** 90% Water and **(F)** 99% Water.

**Table S6**: Details of DLS measurements for **SC7**.

| **Sample** | **Peak 1 Size (nm)** | **Peak 2 Size (nm)** | **PDI** |
| --- | --- | --- | --- |
| **SC7 (10% water : 90% THF)** | **0.9019** | **206.9** | **0.990** |
| **SC7 (30% water : 70% THF)** | **1.918** | **243.0** | **0.634** |
| **SC7 (50% water : 50% THF)** | **1.928** | **229.5** | **0.416** |
| **SC7 (80% water : 20% THF)** | **0.8859** | **94.59** | **0.241** |
| **SC7 (90% water : 10% THF)** | **-** | **620.2** | **0.171** |
| **SC7 (99% water : 1% THF)** | **-** | **573.8** | **0.174** |

**Table S7.** Details of DLS measurements for **RC7**.

| **Sample** | **Peak 1 Size (nm)** | **Peak 2 Size (nm)** | **PDI** |
| --- | --- | --- | --- |
| **RC7 (10% water : 90% THF)** | **2.865** | **214.2** | **1.000** |
| **RC7 (30% water : 70% THF)** | **1.854** | **181.2** | **0.685** |
| **RC7 (50% water : 50% THF)** | **1.895** | **203.8** | **0.210** |
| **RC7 (80% water : 20% THF)** | **-** | **235.0** | **0.219** |
| **RC7 (90% water : 10% THF)** | **-** | **334.4** | **0.214** |
| **RC7 (99% water : 1% THF)** | **-** | **352.6** | **0.199** |

**Table S8.** Details of DLS measurements for **SRC7**.

| **Sample** | **Peak 1 Size (nm)** | **Peak 2 Size (nm)** | **PDI** |
| --- | --- | --- | --- |
| **SRC7 (10% water : 90% THF)** | **0.8411** | **-** | **1.000** |
| **SRC7 (30% water : 70% THF)** | **1.796** | **228.3** | **0.242** |
| **SRC7 (50% water : 50% THF)** | **1.861** | **167.9** | **0.290** |
| **SRC7 (80% water : 20% THF)** | **-** | **183.8** | **0.330** |
| **SRC7 (90% water : 10% THF)** | **-** | **232.7** | **0.192** |
| **SRC7 (99% water : 1% THF)** | **-** | **311.0** | **0.182** |

**Figure S74:** FESEM analysis of (A) **SC7** in 50% water-THF medium , (B) **SC7** in 99% water-THF medium , (C) **RC7** in 50% water-THF medium , (B) **RC7** in 99% water-THF medium.
